# Distinct brain morphometry patterns revealed by deep learning improve prediction of aphasia severity

**DOI:** 10.21203/rs.3.rs-3126126/v1

**Published:** 2023-07-03

**Authors:** Alex Teghipco, Roger Newman-Norlund, Julius Fridriksson, Christopher Rorden, Leonardo Bonilha

**Affiliations:** 1 University of South Carolina; 2 Emory University

## Abstract

Emerging evidence suggests that post-stroke aphasia severity depends on the integrity of the brain beyond the stroke lesion. While measures of lesion anatomy and brain integrity combine synergistically to explain aphasic symptoms, significant interindividual variability remains unaccounted for. A possible explanatory factor may be the spatial distribution of brain atrophy beyond the lesion. This includes not just the specific brain areas showing atrophy, but also distinct three-dimensional patterns of atrophy. Here, we tested whether deep learning with Convolutional Neural Networks (CNN) on whole brain morphometry (i.e., segmented tissue volumes) and lesion anatomy can better predict which individuals with chronic stroke (N=231) have severe aphasia, and whether encoding spatial dependencies in the data might be capable of improving predictions by identifying unique individualized spatial patterns. We observed that CNN achieves significantly higher accuracy and F1 scores than Support Vector Machine (SVM), even when the SVM is nonlinear or integrates linear and nonlinear dimensionality reduction techniques. Performance parity was only achieved when the SVM was directly trained on the latent features learned by the CNN. Saliency maps demonstrated that the CNN leveraged widely distributed patterns of brain atrophy predictive of aphasia severity, whereas the SVM focused almost exclusively on the area around the lesion. Ensemble clustering of CNN saliency maps revealed distinct morphometry patterns that were unrelated to lesion size, highly consistent across individuals, and implicated unique brain networks associated with different cognitive processes as measured by the wider neuroimaging literature. Individualized predictions of severity depended on both ipsilateral and contralateral features outside of the location of stroke. Our findings illustrate that three-dimensional network distributions of atrophy in individuals with aphasia are directly associated with aphasia severity, underscoring the potential for deep learning to improve prognostication of behavioral outcomes from neuroimaging data, and highlighting the prospective benefits of interrogating spatial dependence at different scales in multivariate feature space.

## 1. Introduction

Aphasia is a language processing disorder that often results from strokes affecting the brain hemisphere dominant for language. It is estimated that aphasia impacts around 30% of all individuals who have survived a stroke^1^ and although many individuals experience spontaneous recovery, chronic language impairments are common^2,3,4^, with deficits persisting beyond 6 months in up to 60% of patients^5^. Chronic aphasia is strongly associated with reduced quality of life, more so than stroke alone^6^, Alzheimer’s, or cancer^7^.

Worse aphasic symptoms in the chronic stages have been associated with worse aphasia in the acute period^8^ as well as larger lesion volumes and older age at time of stroke^9,10,11^. Nonetheless, comprehensive models that include lesion characteristics, acute aphasia severity, age and/or demographic information only explain roughly 50% of the variance in chronic aphasia severity^10^. This highlights the significant role of unidentified neurobiological factors in personalized chronic aphasia trajectories. Understanding these elements can enhance predictive models of language impairments and enrich the comprehension of mechanisms that underlie individual aphasia variability.

Beyond lesion size, the spatial location of stroke injury is predictive of aphasia severity. However, modern theoretical models of aphasia neurobiology suggest that language recovery is contingent on the degree of preservation of hierarchically organized neuroanatomical systems beyond the lesion^12–16^. Within this framework^12^, chronic aphasia tends to be less severe when core language specific regions, which support most of recovery, are partially preserved. When language specific areas are not spared, multimodal domain general regions provide the main substrate for recovery at the cost of reduced likelihood of substantial recovery. Finally, when multimodal regions are not intact, recovery depends on left hemisphere regions primarily associated with other functions, or on regions of the contralateral hemisphere, but likelihood of more severe symptoms in the long term is higher.

Despite growing recognition of the importance of spared regions beyond the lesion, our models for understanding chronic aphasic symptoms have not consistently accounted for the contribution of regional or global brain integrity. Nonetheless, aphasia is commonly associated with atrophy in specific regional networks. For example, Egorova-Brumley et al.^17^ reported that individuals with post-stroke aphasia have more atrophy of the inferior frontal gyrus. Stebbins et. al.^18^ demonstrated that cognitive impairments after stroke were associated with gray matter atrophy in the thalamus, cingulate gyrus and widespread distributed regions across frontal, temporal, parietal, and occipital lobes among stroke survivors with or without mild aphasia. Lukic et al.^19^ also demonstrated that preservation of right hemisphere volumes in the temporal gyrus and supplementary motor areas were associated with better language comprehension and production scores, respectively, among individuals with aphasia. Collectively, these findings suggest that brain tissue integrity beyond the lesion is prevalent and potentially closely linked to the severity of chronic aphasia. However, its significance has only recently begun to receive more serious consideration.

**Figure 1.**
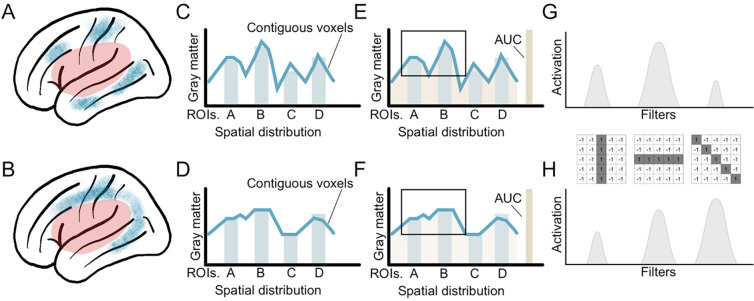
Potential advantages of Convolutional Neural Networks (CNN) for the detection of spatially related changes in brain structure. **Panels A** and **B** provide examples of two patterns of gray matter atrophy in perilesional brain areas (shown in blue) of individuals with identical lesions (shown in red). Despite the average voxel-wise atrophy in brain regions being comparable (bars in **panels C** and **D**), the voxel-wise distribution of the location of atrophy is different, as demonstrated by the lines in **panels C** and **D** indicating contiguous voxel-wise levels. The total atrophy (area under the curve - AUC in **panels E** and **F**) is similar as well. While the differences in the pattern of atrophy are visually intuitive (e.g., based on the shape of atrophy in **panels A** and **B**), they are not captured by conventional statistical models or machine learning approaches. CNNs are ideally equipped to identify such patterns through the application of spatial filters and the identification of motifs based on the shape and spatial dependence of imaging features. For example, each filter can represent a spatial contrast, and each contrast can be more or less represented within each pattern of atrophy (**panels G** and **H**).

A potential explanation for the relative paucity of studies testing the importance of brain integrity in aphasia are limitations concerning methods that can permit the assessment of the spatial distribution of brain atrophy. Indeed, brain atrophy is known to occur within specific patterns across several neurological conditions, for example, Alzheimer’s disease, frontotemporal dementia, epilepsy, among others^19–21^. In these conditions, specific spatial distributions of atrophy, often affecting the same brain regions, are commonly associated with worse symptoms and outcomes. Such patterns remain largely unmapped in the context of stroke. The emergence of artificial intelligence methods capable of extracting information about *spatially dependent* multivariate features from three-dimensional images affords an unparalleled window into the variance in chronic aphasic symptoms that so far remains unexplained by permitting more robust exploration of heterogenous distributions of brain atrophy in individuals (Figure 1).

Recent work has indicated CNNs can adequately discriminate tissue injury patterns that are not detectable with the same accuracy by conventional machine learning methods^22^. Critically, tissue damage in strokes associated with aphasia typically follows the anatomical distribution of middle cerebral artery (MCA) perfusion territories. While there is some interindividual variability in MCA perfusion territory anatomy, many multimodal brain regions such as those in interhemispheric territory (e.g., cingulate cortex, supplementary motor areas) or medial temporal regions (e.g., hippocampus, entorhinal and perirhinal cortices) are spared by MCA or anterior circulation strokes. The degree of preservation of these regions is therefore independent from lesion boundaries. To the extent of our knowledge, no studies have used CNNs for subtyping brain tissue integrity patterns in individuals with chronic aphasia.

**Figure 2.**
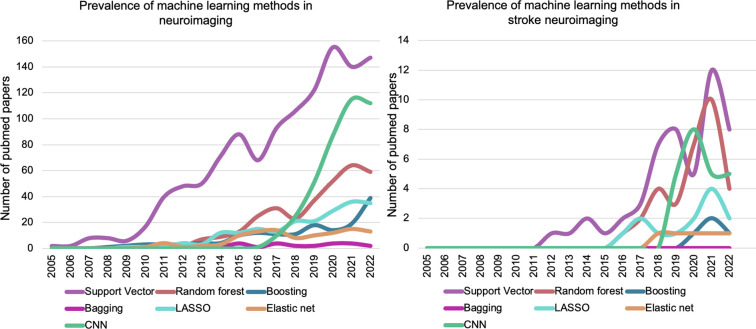
Prevalence of different machine learning methods in neuroimaging more broadly (left) and in neuroimaging of stroke patients (right). The number of publications using different machine learning methods were retrieved from PubMed (y-axis) with the search query “neuroimaging” [AND] the method specified in the legend. In the panel on the right an additional phrase was added to the search: [AND] “stroke”. Both plots show that while support vector machines remain the most prevalent machine learning approach in studies, convolutional neural networks (CNNs) are becoming increasingly more frequently used and oftentimes are used more frequently than other classical machine learning methods, which include bagging and boosting of weaker learners in general, bagging of decision trees specifically (i.e., Random Forest), and linear regression with L1 (LASSO) or L1 and L2-norm penalties (Elastic Net).

CNNs are increasingly applied to neuroimaging studies, including those in stroke, and are already more prevalent than many classical machine learning methods (Figure 2). In the stroke literature, CNNs have been mainly used for lesion segmentation^23–27^. For example, Figure 2 shows 19 studies in the PubMed database retrieved for the terms, “neuroimaging”, “stroke”, and “convolutional neural network”. Excluding review papers, a study actually treating Alzheimer’s patients and a study that did not use neuroimaging data for modeling, the majority of retrieved studies used CNNs for detecting stroke, segmenting lesions, white matter hyperintensities, or other regions of the brain that may be affected by atrophy^28–39^ (N = 12; see Figure S7 and Table S3). Two studies relied on CNNs to accelerate sequence acquisition or increase sequence resolution^40,41^, and another two studies leveraged CNNs for outcome prediction, but in the acute setting^22,42^. Nishi and colleagues^42^ used a CNN to predict good outcome on the Rankin scale of disability after stroke using Diffusion Weighted Images, finding that the CNN outperformed logistic regression applied to the same data as well as a linear regression that was trained purely on lesion size. Karakis and colleagues^22^ report similar findings, showing that a CNN outperformed other classical machine learning methods for predicting type of upper limb motor impairment in stroke using multimodal neuroimaging data. Both studies demonstrate the potential benefits of considering spatial dependence in whole brain neuroimaging data when making predictions about stroke.

In this study, we formulated two main hypotheses. First, we proposed that a CNN applied to maps of brain lesions and overall brain tissue could outperform standard multivariate machine learning methods for predicting aphasia severity by directly modeling three-dimensional brain imaging data. Second, we hypothesized that the CNN would take advantage of spatially dependent neuroanatomical information extending beyond the lesion itself. In essence, we anticipated that the CNN would be able to identify subtle patterns of atrophy across the neural network, which may be crucial in understanding stroke recovery and aphasia progression. CNNs could thus reveal latent or hidden patterns compatible with the quantitative literature that are difficult to detect with traditional methods.

To verify these hypotheses, we develop models to compare CNN with a state-of-the-art and well-established machine learning algorithm (Figure 2), the Support Vector Machine. We contrast these models’ predictive accuracies and investigate their saliency maps, which attribute the spatial patterns driving model prediction (see Figure 3 for analysis overview). Through this investigation, we hoped to illustrate the potential of CNNs for advancing our understanding and prediction of aphasia severity following stroke.

## 2. Methods

### Participants

Two-hundred and thirteen individuals (age = 57.98 +/− 11.34, 62% male) with chronic left strokes that participated in studies conducted at the Center for the Study of Aphasia Recovery (C-STAR) were analyzed retrospectively in the present work. This cohort represents participants that have been seen at the center through 2022. Data was collected at the University of South Carolina and Medical University of South Carolina. All participants gave informed consent for study participation and the study was approved by the Institutional Review Boards at both institutions. Only neuroimaging and behavioral data from participants’ first visits was utilized where longitudinal data was collected. All participants had both behavioral and imaging data available for analysis.

### Behavioral Assessment

Each participant was administered the Western Aphasia Battery-Revised (WAB-R)^43^. The WAB-R comprises multiple subtests for language impairment in aphasia. The current study utilized the aphasia quotient, which collapses spontaneous speech fluency, auditory comprehension, speech repetition and naming subtest performance into one global score that scales between 0 (reflecting worst aphasia impairment) and 100 (reflecting no aphasia impairment). According to the WAB-R, aphasia severity can be classified into 4 categories using the aphasia quotient: very severe (0–25), severe (26–50), moderate (51–75), and mild (>76) aphasia. In the present study, we aimed to identify patients with severe aphasia (WAB-AQ below 50), who comprise 35% of the participant cohort and fall under the “very severe” or “severe” WAB-R categories. Classifiers were tasked to discriminate participants with severe aphasia from all others (i.e., those with “moderate” or “mild” aphasia according to WAB-R). A histogram of WAB-AQ scores across participants is presented in Figure 1B. Neuroimaging data was collected within 10 days of evaluation on the WAB-R.

### Imaging data

Magnetic Resonance Imaging (MRI) was performed at the University of South Carolina or Medical University of South Carolina using a Siemen’s 3T Prisma^44^ equipped with a 20-channel RF receiver head/neck coil. T1 and T2-weighted structural scans were utilized in the current study. A high-resolution T1-weighted MPRAGE sequence was acquired (matrix = 256 × 256 mm, repetition time = 2.25 s, echo time = 4.11 ms, inversion time = 925 ms, flip angle = 9°, 1 × 1 × 1 mm, 192 slices) with parallel imaging (GRAPPA = 2, 80 reference lines). Three-dimensional (3D) T2-weighted sampling perfection with application-optimized contrasts using different flip-angle evolution (SPACE) was used to acquire T2-weighted sequences (matrix = 256 × 256 mm, repletion time = 3200 ms, echo time = 567 ms, flip angle = variable, 1 × 1 × 1 mm, 176 slices) with parallel imaging (GRAPPA = 2, 80 reference lines).

### Image preprocessing

Lesions were segmented manually using T2-weighted images in MRIcron. That is, lesions were drawn by a neurologist (L.B.) or by a researcher with extensive experience with brain imaging in stroke populations. Both were blinded to behavioral assessments. Lesion masks were resampled to the T1-weighted images using nii_preprocess (https://github.com/rogiedodgie/nii_preprocess/tree/v1.1) and SPM8^45^, then refined for any necessary corrections in the case that any additional information about lesion extent was revealed by the T1-weighted image. Anatomical deformation during normalization in the presence of large lesions was avoided using enantiomorphic healing^46^. In this procedure, the lesion boundary is smoothed and the brain tissue inside the smoothed lesion mask is replaced by intact contralateral tissue, thereby exploiting the natural symmetry of the brain to minimize displacement of voxels relative to other methods when normalizing large unilateral lesions^47^. Healed T1 scans were segmented (binary) into volumes containing white matter, grey matter, and cerebrospinal fluid using FAST^48^. The same healed T1 scans were normalized to the MNI152 (2mm) template distributed with FMRIB Software Library (FSL)^49^ using the fsl_anat pipeline (http://fsl.fmrib.ox.ac.uk/fsl/fslwiki/fsl_anat), which combines linear and nonlinear registration methods. Segmented tissue and lesion maps were transformed to template space using k-nearest neighbor interpolation and merged to generate ordinal morphometric maps with voxels inside the lesion being assigned a 4^th^ “tissue” value. This procedure yielded a tissue map with brain structures outside of the lesion and the lesion map itself. Note that the enantiomorphic healed tissue was not included, but it was replaced by the lesion. The maps were then downsampled to 8 mm voxel size and the field of view was cropped to remove any empty space present across all study participants. Finally, each map was scaled to range between −1 and 1. A visual overview of image preprocessing can be found in Figure 3A.

### Cross validation

The partitions that we used for training, tuning, and testing deep and classical machine learning models were preallocated to facilitate more equitable paired comparisons (i.e., t-tests) of performance across 20 repeats of the cross-validation procedure. Repeating cross validation captures the influence of data partitioning noise on the model, which can have substantial impact on performance estimates^50^. Cross validation was stratified to address minority class representation in the partitions. Although we were interested purely in discriminating patients with severe aphasia, the more granular WAB-R aphasia categories were used for stratification, ensuring that partitions sampled data with more similar distributions of raw aphasia quotient *scores* to hedge against the possibility that the model would over or underfit to more specific severity subtypes. Partitions used to tune models were nested to estimate model generalizability more reliably and mitigate the risk of overfitting^51–53^. Six outer folds were used to test model performance, resulting in training sets that contained ~192 patients and test sets that contained ~38. Eight inner folds were constructed to tune the models. More inner folds were selected to improve the likelihood of model convergence on an optimal hyperparameter set. Hyperparameters were tested by grid search, using the smallest mean loss over inner folds as the selection criterion. Models were retrained on the entire outer fold using the selected hyperparameters to maximize data exposure. For deep learning, retraining entailed partitioning the outer training set into one training (70%; N=~135) and validation (30%; N~=58) split, ensuring the test set remains unseen while making validation data available to determine when to cease training.

### Model evaluation

Model performance was captured with precision, F1 scores, and individual as well as balanced class accuracies. These measures were computed by concatenating predictions across outer folds and comparing them to true labels. Individual class accuracies represented the proportion of true positives to false positives with respect to each of the two classes (i.e., severe and nonsevere aphasia). As our task amounts to binary classification, model recall can be reduced to accuracy for the severe class. Accuracy is a highly intuitive measure of model performance but skewed in the presence of class imbalance. For this reason, we computed the mean of individual class accuracies (i.e., balanced accuracy). Model precision captures the fraction of correctly predicted severe aphasia cases (i.e., true positives) out of all severe aphasia predictions made by the model (i.e., true positives and false positives). The F1 score, which represents the harmonic mean between precision and recall, was prioritized above other measures for model assessment. This score is more appropriate when classes are imbalanced, and when it is crucial for the model to optimize for both false positives and false negatives. By seeking a balance between these error types, the F1 score ensures that the model does not favor one class over the other. Preference for the F1 score is motivated by the utility of prognosticating patients with severe aphasia, which has the potential to guide more efficient distribution of clinical resources. In this context, false negative predictions are arguably worse than false positives because patients that could benefit from treatment may be missed. Consequently, we report precision and individual class accuracies to provide better insight into model behavior, but primarily assess models based on F1 scores and, to a lesser extent, consider averaged class accuracies.

### Deep learning

Convolutional Neural Networks (CNNs) are designed to learn a hierarchical representation of the data through a series of connected convolutional layers. These layers learn the weights for small filters that slide over the data through backpropagation, extracting relevant features. Blocks of convolutional layers are broken up by pooling layers that reduce the learned feature map dimensionality, increasing computational efficiency of the network and helping to mitigate the risk of overfitting^54^. The network terminates with fully connected layers that transform features into lower dimensional representations and finally into predictions. CNNs are particularly well-suited for neuroimaging data because they do not require flat inputs like other networks and can be extended to use 3D convolutions that are readily applicable to volumetric data. Although some classical machine learning methods have embedded feature selection (e.g., LASSO), CNNs can be more robust in higher-dimensional settings, learn increasingly more sophisticated representations of the data, learn representations that are spatially invariant, and allow the model to account for spatial dependencies (i.e., consider relationships between neighboring voxels at different scales during learning)^54^.

We used a single-channel 3D CNN to predict patients with severe aphasia by minimizing binary cross entropy loss weighted by inverse class frequencies to compensate for class imbalance. The network was structured based on VGG (Visual Geometry Group) architecture, using small 3×3 convolutional filters, max pooling, and a deep stack of convolutional layers. This kind of architecture has been shown to perform as well or better than alternatives in similar tasks to ours^55^. During tuning, we systematically experimented with 4 levels of network “complexity”, allowing the number of convolutional layers and their channel configuration to vary. The least complex CNN structures contained 4 blocks of convolutional layers and the most complex contained 5 blocks. Each block was followed by a max pooling layer with the number of convolutional layers within blocks varying between 1 and 4 and the number of channels in each layer varying between 8 and 128. Networks terminated with three fully connected layers. The first layer doubled the number of channels in the last convolutional layer, the second layer halved the number of channels and the last layer corresponded to the number of classes.

Aside from batch normalization after the first fully connected layer, network regularization was addressed by: i) adding a dropout layer immediately after and tuning the dropout frequency (0.6, 0.7, 0.8), and ii) tuning an L2-norm penalty applied to the weight parameters (0.001, 0.01). See Figure 3B for full network architecture. Tuning was extended to the network learning rate (0.1e-4, 0.8e-4,1e-4; see supplemental material for decision on magnitude). To speed convergence and prevent suboptimal solutions a cosine annealing scheduler with warm restarts was used to adjust the learning rate^56^. First, the learning rate was reduced using the cosine annealing schedule, decreasing the rate to 1e-10 over 50 epochs. The process was then restarted. The number of epochs over which cosine annealing was scheduled increased by a factor of 2 with each restart. The CNN was trained and tuned over 800 epochs using mini batches of 128 samples to encourage gradient stabilization. See supplemental material for information on measures and criteria used to initiate early stopping during training.

### Classical machine learning

Support Vector Machines (SVMs) were one of the first machine learning methods introduced to neuroimaging^51^ and remain the most popular machine learning method in the field (see Figure 2). We trained SVMs to predict aphasia severity by minimizing hinge loss weighted by inverse class frequencies using the Sequential Minimal Optimization (SMO) algorithm. In SVMs, the kernel trick efficiently transforms data into a higher dimensional space through which a hyperplane maximizing class separability can be more successfully optimized using different kernel functions^57^. Because optimizing more hyperparameters may lead to model overfitting, we assessed more optimistic estimates of SVM generalizability (i.e., performance) by training independent models using the linear and radial basis kernel functions. Indeed, when we tested SVMs that tuned the kernel function alongside other hyperparameters, performance plummeted (see supplemental section). Other hyperparameters were tuned using random search with 300 logarithmically spaced bins for each parameter. These included kernel scale, which controls the smoothness of the kernel function (ranging from 1e-3 to 1e3), and cost, which controls the width of the margin and balances the trade-off between maximizing the margin and minimizing hinge loss (ranging from 1e-3 to 2e4).

We addressed the possibility that poorer SVM performance relative to CNN reflects optimization failure in high dimensional feature space by cross validating two additional SVMs independently, one which used Principal Component Analysis (PCA) as a data preprocessing step and another that relaxed component orthogonality constraints by applying Independent Component Analysis (ICA) to the components. Reducing the number of features in the data prior to training a model for classification or regression is a commonly employed strategy (e.g., principal component regression) and PCA/ICA are often recommended to be used precisely in this way with SVM in neuroimaging^58^. For both dimensionality reduction techniques, model order selection was expressed as a hyperparameter that was tuned in the inner folds by validating SVM models trained on lower dimensional spaces that retained between 1 and 75 components (i.e., 75 total values tested). Dimensionality reduction was folded into cross-validation—test and validation data were always projected into lower-dimensional spaces defined on training data.

### Fusing classical and deep learning methods

We considered that SVMs and CNNs may be sensitive to different patterns in the data in several ways. The simplest approach involved computing a weighted average of model prediction probabilities, programmatically adjusting the weight given to one model over the other and measuring the resulting ensemble’s cross validated performance. The next approach integrated model predictions in a more sophisticated way, tuning a Linear Discriminant Analysis (LDA) to learn the optimal strategy for combining probabilistic model predictions. We report the results for LDA but note that, in our experiments, logistic regression and decision trees performed just as well. Finally, we tested whether prediction performance could be improved by training SVMs to directly exploit the features extracted by CNNs. That is, we cross-validated a SVM that was trained on the CNN’s penultimate fully connected layer (i.e., ~64 features in our case).

We further explored the impact of data dimensionality on SVM performance by cross-validating two additional SVM models, one trained on feature saliency maps generated by deep Shapley Additive exPlanations (SHAP) and another trained on feature maps generated by Gradient-weighted Class Activation Mapping++ (Grad-CAM++). This comparison additionally permitted us to evaluate whether it was likely that the CNN outperformed SVMs by identifying unique patterns in the data unavailable to SVMs. Deep SHAP extends the kernel SHAP framework to deep learning models using an enhanced version of the DeepLIFT algorithm^59,60^. Kernel SHAP leverages Shapley values, which are a game theoretic approach for quantifying the average marginal contribution of a player in a cooperative game^61^. The Shapley value for a feature describes its role in deviating the prediction from the average or baseline prediction with respect to a specific sample in the data. SHAP is an extension of Shapley values that uses the conditional kernel with k nearest neighbors (corresponding to 10% of the samples) for evaluating feature importance^62^. Feature importance is assessed by computing model performance as a function of the inclusion of random subsets of features in the model selected by monte-carlo sampling. The Grad-CAM++ algorithm^63^ improves on the original Grad-CAM method of explaining CNN predictions, which involves computing the weighted sum of the gradients of the predicted class score with respect to the feature maps of the last convolutional layer in the network^64^. The Grad-CAM++ algorithm differs by introducing a second-order term into the computation, taking into account the curvature of the CNN decision boundary with respect to changes in feature maps. Some work has indicated that this can improve the reliability and localization of feature importance, including in MRI data, and particularly in the context of complex or nonlinear decision boundaries^63,65^. Grad-CAM++ is also more robust when the CNN contains dropout layers that contribute to more diffuse gradients because it considers spatial correlation between CNN feature maps to derive importance. Fundamentally, Grad-CAM++ is designed to account for the special spatial properties of CNNs that deep SHAP does not directly consider when estimating feature importance.

Cross validation of the CNN was implemented in PyTorch^66^ and cross validation of the SVM was performed with MATLAB’s machine learning toolbox^67^.

### Subtyping distinct brain patterns through deep learning

Unsupervised learning of Grad-CAM++ maps was used to explore the heterogeneity of brain patterns learned by the CNN. Feature saliency maps were used as inputs instead of CNN layers to understand which brain structures were implicated. Reliable clustering solutions were identified and generated through the framework of consensus clustering, wherein a resampling technique is used to compute clustering consensus^68^. Consensus is defined as the proportion of times that a pair of samples from the data are placed into the same cluster when they are subsampled together. This measure is computed for each pair of samples in the data to form a symmetric similarity matrix that embeds a consensus solution, which can be retrieved using a similarity-based clustering algorithm^69–72^. The distribution of values within a consensus matrix can be additionally used to understand the internal validity of a solution and used for model-order selection (see supplemental material)^70,73^.

Here, we subsampled 60% of the voxels in feature saliency maps 1000 times, each time performing k-means clustering using the k-means++ algorithm with 250 replicates to improve solution stability^74^. The eta^2^ distance measure was used for k-means clustering (i.e., 1 minus the eta^2^ coefficient). This coefficient aims to capture voxelwise similarity between whole brain maps, with 1 reflecting identical maps, and 0 reflecting maps that share no variance^75^. It shares some resemblance to the Pearson correlation coefficient but quantifies similarity on a point-by-point basis, therefore accounting for scaling and offset when comparing two whole brain maps^75^. For each subsample, clustering was repeated to generate solutions ranging from 3 to 30 clusters. The most complex solution (i.e., highest number of clusters) that exhibited high reliability was selected. Briefly, Hartigan’s dip test of unimodality was used to exclude solutions that showed significantly unimodal consensus distributions (i.e., no consensus because samples were not consistently being placed in either the same or different clusters). The proportion of ambiguously clustered pairs^73^ was then used to approximate the reliability of the remaining solutions (see supplemental material). Clusters for the selected solution were extracted by applying affinity propagation^76^ to the corresponding consensus matrix. Affinity propagation is an exemplar-based clustering approach that has been shown to be less sensitive to initialization parameters than other similar methods^76,77^. Critically, an exemplar-based approach provided us a representation of each cluster.

### Decoding morphometry patterns

Subgroup-representative feature saliency maps were decoded to probe for functional associations of morphometry patterns predictive of aphasia. Meta-analyses were generated for 200 topics of an author-topic model of the neuroimaging literature^78^. Note, we used the 7^th^ version of the model retrieved with NiMARE^79^. Meta-analyses for the topics were generated with neurosynth^80^. Briefly, in this framework, meta-analysis is performed by separating all studies into two groups: those that were associated with a topic and those that were not. Next, a search was performed for voxels where activity was more consistently reported in the topic-associated set of studies. This involved extracting the activation tables from these two groups of studies, creating contingency tables at each voxel that described whether activity was present and whether the topic was associated, and then performing a chi-square test. Decoding was performed by computing the Pearson correlation coefficient between each topic meta-analysis and the saliency map associated with each subgroup after removal of saliency values within the lesion. Topics with a Bonferroni corrected p-value < 0.0001 and correlation above 0.2 were retained.

## 3. Results

### Evaluating the quality of CNN model predictions and the consistency of learned features

We first evaluated how well a CNN can predict patients with severe aphasia from morphometry and lesion maps. Distributions of CNN model performance over 20 repeats of cross-validation indicated the model achieved moderate-to-high accuracy (Figure 3A). The median accuracy for severe cases of aphasia was 0.88 (ranging 0.81 to 0.93) and the median of accuracy averaged across the two classes was 0.77 (ranging 0.74 to 0.78), demonstrating that the model was very accurate at identifying severe aphasia and generally accurate at predicting aphasia severity. The model performed less well at identifying patients with nonsevere aphasia, attaining a median nonsevere class accuracy of 0.67 (ranging 0.61 to 0.69). Precision reflects the same result, highlighting a slight tendency towards false positives to achieve high prediction accuracy for patients with severe aphasia. The precision score of 0.59 (ranging 0.55 to 0.6) indicated that when the model predicted a patient had severe aphasia, it was correct 59% of the time. The model’s median F1 score was 0.7 (ranging 0.67 to 0.72) illustrating that it achieved a good balance between precision and recall. For reference, a model that always guesses the majority class would achieve an F1 score of 0 and one that always guesses severe aphasia would achieve 0.52. We reiterate that our evaluation of models favored the F1 score, and to a lesser extent, balanced accuracy (see methods).

The robustness of the CNN classifier was tested more formally. Class labels were randomly permuted 500 times and the model building and testing process was repeated. Our results showed that CNNs trained on permuted data produced a distribution of F1 scores with no overlap with CNNs trained on unpermuted data (Figure 3B). Performance significantly better than chance (p < 0.000001) indicated the network learned meaningful features that distinguish aphasia severity. As further evidence of model robustness, we showed that learned features exhibited a similar structure across cross-validation repeats. T-distributed stochastic neighbor embedding^114^ was used to group participants based on features extracted from all models and revealed distinct clusters for predicted classes (Figure 3C; also see supplemental material). The influence of lesion size (i.e., small, medium and large based on 3 quantiles) and more granular aphasia severity categories on the model decision boundary were also investigated (Figure 3C). CNN models often correctly classified participants with medium-sized lesions that belonged to both severity classes, suggesting lesion size was unlikely to be the sole driver of predictions. Models often predicted nonsevere aphasia when participants had small lesions and severe aphasia when participants had large lesions, reflecting the established relationship between larger lesions and higher severity. Most misclassifications involved participants just below the severe aphasia boundary.

### Evaluating the quality of CNN model predictions against classical machine learning

**Figure 4.**
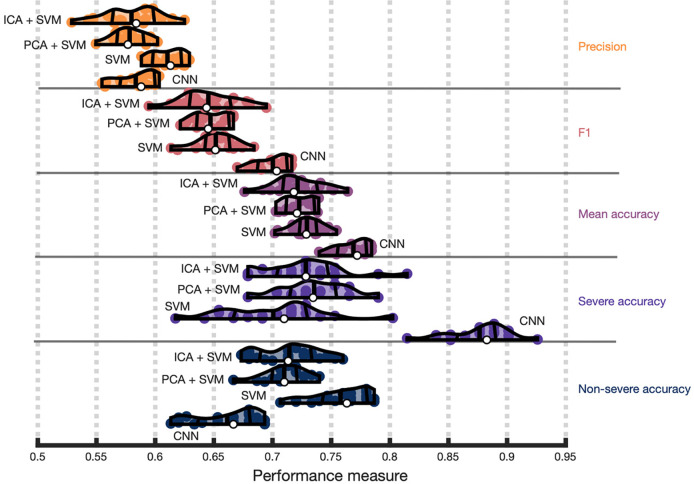
Convolutional Neural Networks (CNNs) outperform classical methods. Violin plots showing CNN and Support Vector Machine (SVM) performance across 20 repeats of the nested cross-validation scheme. Performance is presented in terms of individual class accuracies (severe and nonsevere accuracy), weighted accuracy (average across the two classes), F1 scores, precision, and recall. Violin plot colors correspond to different performance measures which are additionally separated by horizontal lines. Within each performance measure the first or topmost violin shows the performance of a SVM combined with an ICA preprocessing step, the following violin plot shows the performance of a SVM combined with a PCA preprocessing step, the penultimate violin plot shows the performance of a SVM without dimensionality reduction as a preprocessing step, and the final violin plot depicts CNN performance as a baseline (i.e., from Figure 2). See previous figure for information represented in each violin plot.

We next determined whether SVMs outperformed CNNs (Figure 4). This could indicate that CNNs, which generally demand more computational resources and involve adjusting more parameters, may have difficulties with overfitting to our sample size and type of data. We first noticed that linear SVMs outperformed nonlinear SVMs across repeats and focus on these models for brevity (Figure S1). Paired t-tests comparing model performance across repeats revealed that linear SVMs had significantly worse F1 scores (M=0.65, SD=0.02) compared to CNNs (M=0.7, SD=0.01), t(19) = −10, p < 0.000001, Cohen’s d =−2.24. Overall, SVMs mean accuracy was worse (M=0.73, SD=0.01) than CNNs (M=0.77, SD=0.01), t(19) =−9.8, p < 0.00000001, Cohen’s d=−2.2. Although we focus on F1 and balanced accuracy throughout the results, our supplemental material extends the same analyses to other measures, which we display in figures throughout to provide a more comprehensive view of model performance.

PCA and ICA were inserted into the SVM model building process to address the possibility that SVMs performed worse only as a consequence of the high dimensionality of our data (Figure 4). Generally, we found that linear SVMs again outperformed other SVMs in this context and focus on the linear models (Figure S1). Paired t-tests revealed usage of PCA versus ICA conferred no significant performance benefit according to any of the measures (p > 0.4) suggesting ICA did not capture considerable nonlinearities. Further, using dimensionality reduction did not improve SVM performance according to F1 (p > 0.2) or mean accuracy (p > 0.08).

### Fusing CNN and SVM predictions

**Figure 5.**
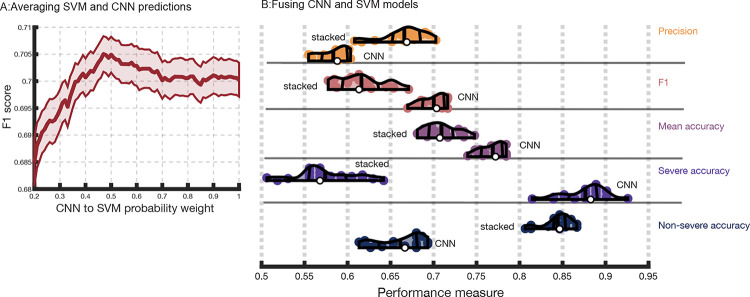
Classical machine learning does not add uniquely predictive information. **Panel A**: F1scores (y-axis) that result when making final test set predictions by averaging the probabilities assigned to classes by the SVM and CNN models. The weight given to the CNN probabilities over SVM probabilities in the weighted average is depicted on the x-axis (i.e., 1 means only CNN probabilities are considered, 0 means only SVM probabilities are considered, 0.5 amounts to averaging the probabilities without any weighting). The thick solid line represents the mean F1 score attained across 20 repeats of the cross-validation scheme. The shaded area corresponds to standard error of the mean. **Panel B:** Violin plots showing CNN performance over 20 repeats of the cross-validation scheme (bottom violins in each row) relative to stacking SVM and CNN predictions (top violin in each row). Violin plot colors correspond to different performance measures which are additionally separated by horizontal lines (i.e., “rows”).

Integrating CNN and SVM models provided us the opportunity to test whether CNNs and SVMs identified unique patterns in the data. First, we averaged probabilistic class predictions from both models to make final predictions. This analysis demonstrated that exhaustively tweaking the weight given to the CNN over SVM predictions in the average did not produce a single model that substantially outperformed the CNN on the F1 score (Figure 5A). Indeed, a paired t-test revealed no significant difference between the maximum attained F1 score from the entire ensemble set (M=0.71, SD=0.15) and the CNN (M=0.7, SD=0.14), t(19) = 2, p = 0.056, Cohen’s d = 0.46. The steep slope of the curve in Figure 5A highlights the considerable extent to which prediction benefitted from the cumulative accumulation of information learned by the CNN.

The possibility that a more complex criterion for fusing models could improve predictions was explored by stacking models with Linear Discriminant Analysis (Figure 5B). Paired t-tests showed that stacking produced significantly worse F1 scores (M=0.62, SD=0.03) than the CNNs (M=0.7, SD=0.01), t(19) = −11.4, p < 0.00000001, Cohen’s d = −2.55. Stacked models had lower mean accuracy (M=0.71, SD=0.02) than CNNs as well (M=0.77, SD=0.01), t(19) = −10.5, p < 0.00000001, Cohen’s d = −2.35. These findings demonstrate that CNNs learned unique morphometry patterns and SVMs did not identify any patterns that could further improve predictions.

### Evaluating the impact of CNN properties on feature importance

Multiple feature saliency mapping methods were used to understand if better CNN performance was the result of the special spatial properties of CNNs. For simplicity, we focused on the mean performing models across repeats of cross-validation but note that learned features exhibited a similar structure across repeats (Figure 3C). Grad-CAM++ and deep SHAP saliency maps generated for CNNs were compared to SHAP saliency maps generated for SVMs. As only Grad-CAM++ maps are explicitly sensitive to CNN spatial properties we expected these maps to look different from SHAP and deep SHAP saliency maps, and for SHAP maps to be more similar to each other, provided that the CNN models exploited spatial dependencies in the data. To facilitate model comparisons, we focused on predictions that were accurate for CNN and SVM models.

First, map similarities were compared using the eta^2^ coefficient (see methods). Similarities were computed between each participant’s SHAP and deep SHAP, SHAP and Grad-CAM++, and deep SHAP and Grad-CAM++ maps. Paired t-tests across participants showed SHAP and deep SHAP maps were significantly more similar to each other (M=0.58, SD=0.08) than Grad-CAM++ maps were to deep SHAP maps (M=−0.37, SD=0.16), t(153) = 92.1, p < 0.0000001, Cohen’s d = 4.5, or to SHAP maps (M=−0.47, SD=0.16), t(153) = 75.9, p < 0.0000001, Cohen’s d = 4.

**Figure 6.**
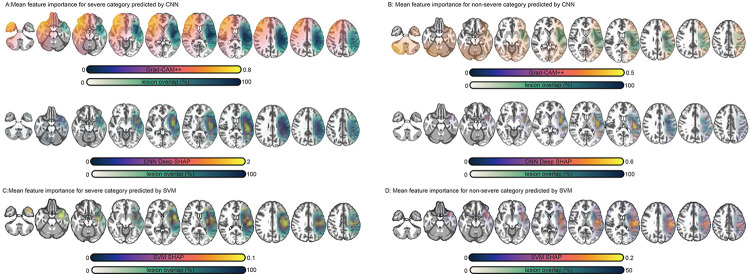
Group-averaged saliency maps. **Panel A:** Montage in the top row shows mean Grad-CAM++ saliency for patients correctly predicted by the CNN to have severe aphasia (purple to yellow) and their lesion overlap in percentage units (white to dark blue) superimposed on a normalized template. Montage in the bottom row shows mean deep SHAP saliency maps for patients correctly predicted by the CNN to have severe aphasia. Negative SHAP values were replaced with zeros to reflect feature contributions only towards the class predicted by the model. Brighter yellow colors reflect higher feature importance and darker blue colors reflect greater overlap of lesions in the patient cohort. The alpha channels for lesion overlap and mean saliency are modulated by the respective values of those maps to highlight differences between maps. **Panel B:** Identical to panel A but this montage shows mean Grad-CAM++ saliency for patients correctly predicted by the CNN to have nonsevere aphasia. **Panel C:** Identical to previous panels but the montage shows mean SHAP saliency for patients correctly predicted by the SVM to have severe aphasia. **Panel D:** Identical to previous panels but the montage shows mean SHAP saliency for patients correctly predicted by the SVM to have nonsevere aphasia.

Group-averaged feature maps confirmed these trends (Figure 6). Grad-CAM++ maps alone highlighted contralateral regions. These were anterior to the concentration of lesions in the group and predicted severe aphasia. Whether predicting severe or non-severe aphasia, SVMs emphasized the region where lesions were concentrated in the group. This was also true of deep SHAP maps generated for CNNs (see Figure S3 for individual maps).

**Figure 7.**
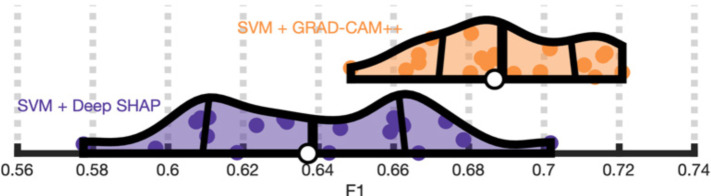
Grad-CAM++ saliency maps capture unique predictive information. **Panel A:** Violin plots showing that a SVM trained on deep SHAP feature saliency maps (purple) attains poorer F1 scores (x-axis) across 20 repeats of the cross-validation scheme than a SVM trained on the Grad-CAM++ saliency maps (purple), which are capable of capturing spatial dependencies exploited by a CNN.

If the unique patterns displayed in Grad-CAM++ maps are meaningful and not an artifact or noise, models trained directly on these saliency maps should outperform models trained on SHAP maps. SVMs trained on Grad-CAM++ (M=0.69, SD=0.02) achieved significantly higher F1 scores than SVMs trained on deep SHAP (M=0.64, SD=0.03), t(19) = 4.5, p < 0.001, Cohen’s d = 1.1 (Figure 7). Remarkably, training SVMs on these higher-dimensional maps resulted in predictions that were as good as SVMs trained on lower-dimensional CNN features (p > 0.1). These SVMs achieved parity with CNN performance (Figure S2).

**Figure 8.**
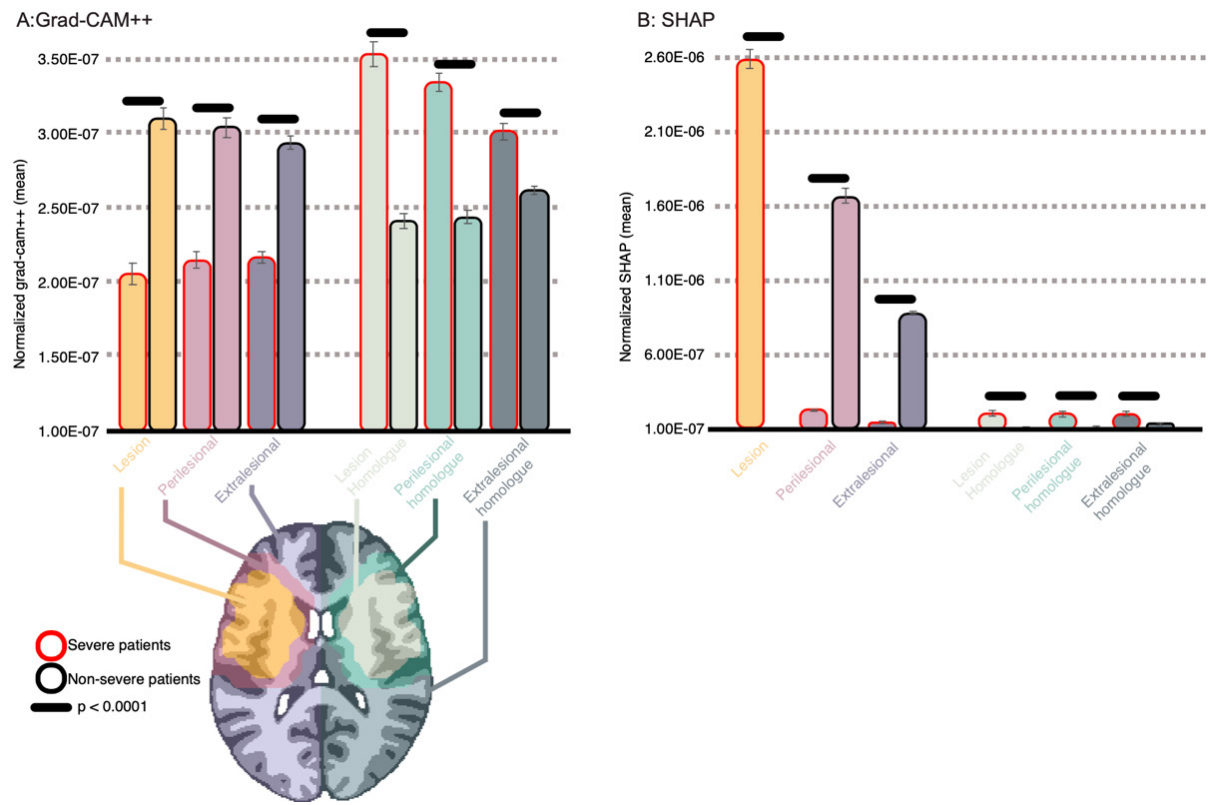
Mean normalized feature saliency within regions of interest. **Panel A:** Grad-CAM++ feature saliency maps for the CNN were normalized to sum to 1 and the mean value was extracted across voxels of 6 regions of interest (see brain image on bottom) independently for patients predicted to have severe aphasia and patients predicted to have nonsevere aphasia: the lesion (orange), the lesion’s right hemisphere homologue (mint), the perilesional area (pink), the perilesional homologue (light green), the extralesional area (i.e., everything in the left hemisphere that’s not part of the lesion or perilesional area; dark blue), and the extralesional homologue (dark green). Mean saliency values (y-axis) are plotted on a bar chart where color corresponds to the region of interest and the outline of the bar corresponds to the predicted class (i.e., red outline is severe and black outline is nonsevere). Error bars represent standard error of the mean. Mean difference between severe and nonsevere patients was tested with two-sample t-tests and horizontal lack lines above error bars indicate significance (p < 0.0001) **Panel B:** Identical to panel A except mean SHAP values (y-axis) are plotted on the bar chart, expressing saliency assigned by the SVM during prediction. As in the previous figure, negative SHAP values were replaced by zeros before normalization.

Having established that the qualitative patterns in Figure 6 are meaningful, we performed a region of interest (ROI) analysis on feature maps normalized to have a sum of 1. This analysis examined whether the models’ focus on lesioned, perilesional and extralesional regions as well as their homologues varied as a function of predicted aphasia severity (see ROI generation methods in supplemental material). Figure 8 shows that Grad-CAM++ saliency was significantly higher in all left hemisphere regions for nonsevere compared to severe predictions based on two-sample t-tests (p < 0.0001). In contrast, saliency was significantly higher in all right hemisphere regions for severe predictions (p < 0.0001). SVM SHAP saliency maps demonstrated higher feature importance in the lesion for severe prediction, but higher importance in the perilesional and extralesional ROIs for nonsevere predictions (p < 0.0001). Feature saliency was low for homologue ROIs across patient class; however, it was relatively higher for severe predictions (p < 0.0001). These patterns further establish that CNNs relied on information outside the lesion for successful prediction.

### Subtyping patterns learned by the CNN

**Figure 9.**
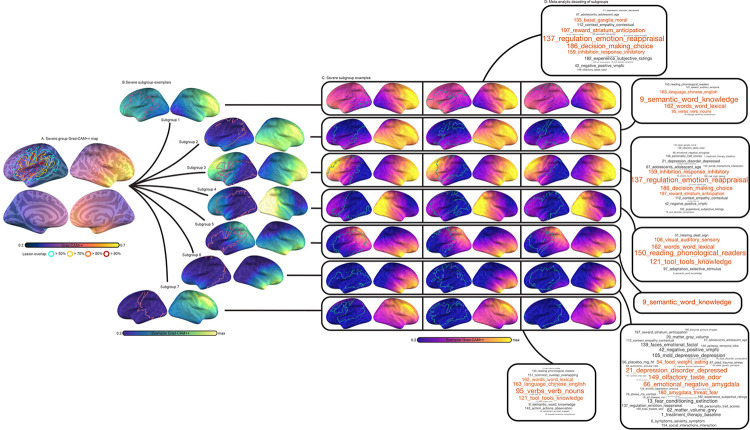
Clustering severe patients using CNN saliency maps. **Panel A:** Group-averaged Grad-CAM++ feature maps (thermal heatmap) with lesion extent superimposed (outline). Lesion extent is shown for the group based on several percentage thresholds of overlap. **Panel B:** Exemplar patients for each patient cluster or “subgroup” are displayed (viridis colormap) along with each patients’ specific lesion map (pink outline). Relative feature importance is shown so the maximum value for each subgroup is different. **Panel C:** Saliency maps for three example individual participants that belong to each subgroup are shown (thermal) with their specific lesion maps (green outline), highlighting consistency in feature importance within subgroups. Volume maps were projected onto the fsaverage surface for visualization using RF-ANTs^81^. **Panel D:** Decoding of subgroup networks (i.e., exemplars) based on Pearson correlation coefficients between extralesional Grad-CAM++ estimates and 200 meta-analyses of topics identified by an author-topic model of the neuroimaging literature. Word clouds show all associated topics with a Pearson correlation above 0.2 (and Bonferroni p < 0.0001). Each topic is named based on the 3 individual neuroimaging terms that load most strongly onto the topic. The index of the topic within the model is shown to facilitate cross-referencing the full set of terms^78^. Word size is modulated by the magnitude of the Pearson correlation coefficient. The top 4 associated topics are shown in red.

**Figure 10.**
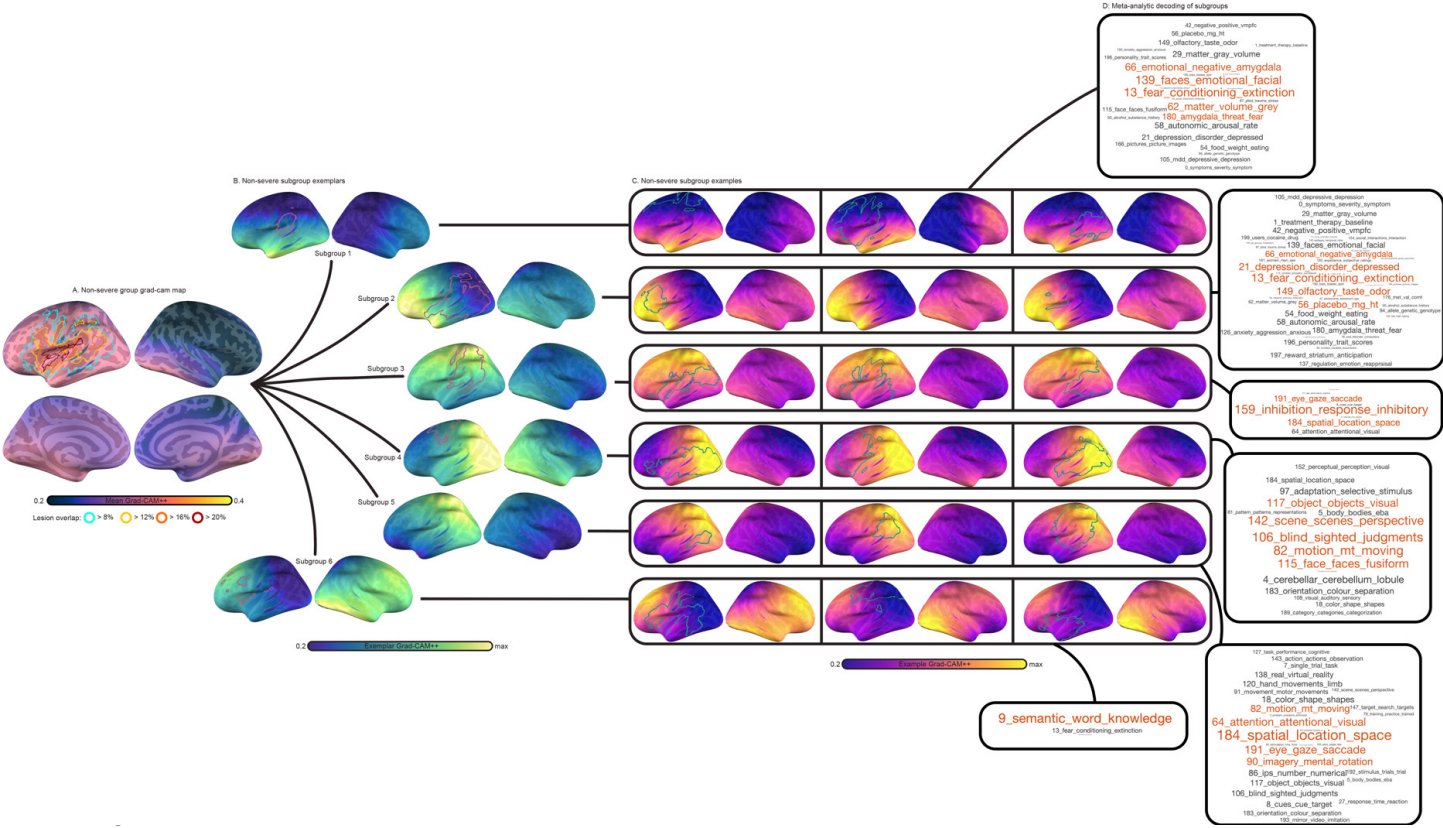
Clustering nonsevere patients using CNN saliency maps. **Panel A:** Group-averaged Grad-CAM++ feature maps (thermal heatmap) with lesion extent superimposed (outline). Lesion extent is shown for the group based on several percentage thresholds of overlap. **Panel B:** Exemplar patients for each patient cluster or “subgroup” are displayed (viridis colormap) along with each patients’ specific lesion map (pink outline). Relative feature importance is shown so the maximum value for each subgroup is different. **Panel C:** Saliency maps for three example individual participants that belong to each subgroup are shown (thermal) with their specific lesion maps (green outline), highlighting consistency in feature importance within subgroups. **Panel D:** Decoding of subgroup networks (i.e., exemplars) based on Pearson correlation coefficients between extralesional Grad-CAM++ estimates and 200 meta-analyses of topics identified by an author-topic model of the neuroimaging literature. Word clouds show all associated topics with a Pearson correlation above 0.2 (and Bonferroni p < 0.0001). Each topic is named based on the 3 individual neuroimaging terms that load most strongly onto the topic. The index of the topic within the model is shown to facilitate cross-referencing the full set of terms^78^. Word size is modulated by the magnitude of the Pearson correlation coefficient. The top 4 associated topics are shown in red.

In a final analysis, we mapped the heterogeneity of integrity patterns learned by the CNN, grouping patients according to the similarity of their feature saliency maps by applying consensus clustering to Grad-CAM++ maps generated for all CNN predictions (see methods and supplemental material). Separate clustering analyses were carried out for severe and non-severe patient predictions. Subsampling the data revealed strong evidence of cluster structure, with solutions containing 7 and 6 clusters for severe and nonsevere saliency maps displaying high reliability and near unanimous consensus according to the proportion of ambiguously clustered pairs (Figure S5). Analysis of similarity between samples of the same cluster and samples belonging to other clusters reinforced that, overall, clustering distinguished highly unique patterns with individuals displaying high similarity to others of the same subtype (Figure S6). Figures 9 and 10C typify these effects, showing morphology patterns within subgroups exhibited high similarity.

Although group-averaged saliency maps showed strong class differences in lateralization (i.e., left hemisphere for nonsevere aphasia and right hemisphere for severe aphasia), subgroups of patients exhibited both lateralization patterns, and most subgroups involved more bilateral patterns than suggested by group effects. For example, subgroups 1, 3 and 5 for individuals with severe aphasia showed moderate-to-high saliency in the right hemisphere despite an overall stronger emphasis on the left hemisphere (Figure 9B). In individuals with nonsevere aphasia, one subgroup showed right-lateralized saliency (subgroup 6) and multiple subgroups showed modest-to-moderate saliency in the non-lateralized hemisphere (subgroups 6, 4, and 2). The right-lateralized subgroup (6) showed remarkable similarity to a subgroup of severe aphasia individuals (2) but was distinguished by more bilateral saliency. Indeed, many subgroups of different classes exhibited overall similar saliency patterns, differing mainly by which hemisphere was more strongly emphasized. As an example, one severe aphasia subgroup (4) displayed highest feature saliency in right temporoccipital cortex, while another nonsevere subgroup (4) displayed peak saliency in left temporooccipital cortex. For a thorough list of similarities see supplemental material. We note that despite having lower importance, features contralateral to the saliency peak contributed meaningfully to model performance. “Ablated” SVMs trained on different hemispheres of saliency maps performed slightly worse than SVMs trained on bilateral maps (Figure S4).

Decoding saliency maps using the wider neuroimaging literature provided evidence that some of the variability in aphasia severity is grounded in damage to different language subsystems, but also to non-language systems outside of the area of stroke, as well as aging (Figure 9,10B,D). First, different portions of the anterior frontal and temporal cortex were associated with different language processes, including semantics (i.e., when anterior temporal pole or posterior inferior temporal gyrus were involved; nonsevere subgroup: 6; severe subgroups: 2,5), reading (i.e., when left posterior temporoccipital cortex was involved; severe:4), spontaneous overt speech (i.e., when left middle frontal gyrus was involved; severe:7), and lexical-semantics (i.e., when all of the aforementioned left hemisphere regions and more of frontal cortex, including inferior frontal gyrus, were involved; severe:9). Notably, severe aphasia was more likely to involve saliency patterns targeting language subsystems (c.f., Figure 9,10D). Second, decoding showed different portions of superior parietal cortex used for prediction were associated with response inhibition, object and spatial processing, including visuospatial memory and working memory (nonsevere:3,5). Third, patterns targeting anterior frontal cortex around the frontal pole and the temporal pole together were associated with higher-level functions such as decision making, but also age, symptom severity, and a range of brain disorders that included epilepsy and Alzheimer’s (severe: 1,3,6; nonsevere: 1,2). For more comprehensive analysis, including details about each meta-analytic topic, see supplemental material.

Finally, we found that patient subgroups were not associated with lesion size, as hinted by the lesion outlines in Figures 9,10. A one-way ANOVA investigating differences among severe patient subgroups was not statistically significant, F(6,108) = 0.77, p = 0.6. An identical effect sought for the nonsevere subgroup was also insignificant, F(5,110) = 0.67, p = 0.65. Accuracy was not statistically different among severe, F(6,108) = 0.53, p = 0.78, and nonsevere subgroups, F(5,110) = 0.46, p = 0.8 (Tables S1, S2).

## Discussion

The integrity of tissue outside of the lesion offers a rich source of information that stands to improve models of stroke outcome. The current study leveraged deep and classical machine learning to understand how well brain morphology and lesion anatomy can predict aphasia severity in chronic stroke. In a repeated, nested, cross-validation scheme, we found that Convolutional Neural Networks (CNNs) performed well on this task, achieving a median balanced accuracy of 77% and a median F1 score of 0.7. This performance was both significantly better than chance and better than Support Vector Machines (SVMs). Using a variety of techniques for fusing the information learned by CNNs and SVMs, we found that SVMs were not sensitive to uniquely predictive information. A thorough investigation of feature saliency confirmed that improved CNN predictions were rooted in the identification of more diverse patterns of brain morphology capitalizing on information outside the area of injury. Clustering the patterns attended by the network showcased diverse aphasia-related atrophy patterns and revealed a considerable degree of individualization.

### Prediction of aphasia severity benefits from deep learning

While CNNs can outperform classical machine learning by detecting patterns that other methods are insensitive to, this is not guaranteed in every application. For example, such patterns might be absent and deep learning can be more likely to overfit in small samples^82^. The current study suggests that in modest samples of patients with chronic stroke, deep learning can be effective. We used nonparametric testing to demonstrate that CNNs made predictions significantly better than chance, showed that they outperformed classical machine learning methods on our task, and demonstrated that they learned features in a consistent way, exhibiting similar feature structure across hundreds of models trained on different data partitions. We attribute this success to the networks’ ability to extract richer latent features from high-dimensional neuroimaging data. Indeed, we found that using more conventional methods of dimensionality reduction, like Principal or Independent Components Analysis, did not necessarily improve performance in classical machine learning. However, classical machine learning performed as well as CNNs when trained on lower-dimensional features learned by the networks or higher-dimensional saliency maps capturing the networks’ attention. These results corroborate and extend recent work demonstrating that CNNs can be successfully deployed outside their current niche in the stroke literature for lesion segmentation and can effectively predict stroke outcomes better than classical methods^22,42^. To the best of our knowledge, this work is the first display of CNN efficacy in chronic aphasia. The relative success we were able to obtain with our model hints at the exciting possibility that with continued development and access to larger and more diverse samples, it may soon be possible to deploy similar models at point-of-care, where prediction of aphasia severity may help healthcare professionals prepare patients for their anticipated outcomes and inform interventions.

### Evidence of improved prediction through consideration of spatial dependence

The current study used CNNs based on the understanding that they can provide a novel window into brain morphometry outside the lesion that is predictive of aphasia severity. The first line of evidence indicating that our CNNs exploited spatial properties was that they outperformed SVMs, which were not able to provide any unique predictive information. Feature saliency maps produced more compelling confirmation. Group-averaged saliency maps that utilized a method explicitly sensitive to CNN spatial properties (Grad-CAM++) highlighted markedly different features than methods that were insensitive to these properties (SHAP). Compared to SHAP, Grad-CAM++ emphasized an entirely different hemisphere in patients with severe aphasia and showed a high degree of individualization, highlighting regions distal to the lesion, in patients with nonsevere aphasia. Critically, SHAP saliency maps were exceedingly similar between CNNs and SVMs, presenting strong evidence that CNN performance was related to exploitation of spatial information. These patterns were corroborated by more quantitative regional analyses, suggesting that often-overlooked spatial dependencies in neuroimaging data may be critical for building more predictive multivariate models.

Post-hoc attribution of attention for CNNs is an active area of research that has seen a proliferation of methods capable of producing slightly different explanations of the model’s decision^83,84^. It’s critical to note that these methods can be unreliable and there is no single recommendation for all networks^85^. Nevertheless, our results indicated Grad-CAM++ can provide useful explanations. In our data, we found that a linear SVM trained on Grad-CAM++ attributions could achieve performance statistically indistinguishable from the CNN and significantly better than other SVM models trained on raw or dimensionality-reduced (PCA, ICA) data. The capacity of these saliency maps to predict aphasia severity as well as the latent CNN features provides independent support for their credibility.

### Hemispheric trends in morphometry predictive of aphasia severity

Our findings converged on specific features associated with aphasia severity. Identification of individuals with severe aphasia relied on features contralateral to the lesion, whereas identification of nonsevere aphasia relied on ipsilateral features. This global pattern of *relative* saliency illustrates that the CNN identified atrophy in the right hemisphere as a strong predictor of severe aphasia. In the absence of clear right hemisphere atrophy, when aphasia was less severe, the network focused on atrophy of left hemisphere regions. In contrast, the SVMs focused on and around the lesion, reflecting to some extent, attention to lesion size. Thus, the discrepancy between CNN and SVM performance signifies the modest but significant gain associated with consideration of morphology patterns outside of the lesion.

Attention to right hemisphere atrophy is consistent with contemporary models of aphasia recovery, which tend to emphasize worsening aphasia severity as damage spills out of core language regions and into non-language left hemisphere regions, and finally into right hemisphere regions^12,86,87^. This view offers a potential reconciliation of conflicting evidence about the contribution of the right hemisphere during language recovery as measured by functional neuroimaging studies^88^ by proposing that worse outcomes are the consequence of more extensive left hemisphere damage that necessitates atypical involvement of the intact regions, which happen to be in the right hemisphere. Our findings demonstrate that individuals with severe aphasia additionally have extensive atrophy of the right hemisphere.

These results contribute to a growing appreciation of extralesional tissue integrity as an independent source of variance in aphasia. It is well-established that coarse information about lesion location and size explains a large amount of variance in acute and chronic stroke outcomes, including aphasia severity^9,11^. At the same time, stroke injury can trigger processes that have a widespread impact on the brain (e.g., neuroinflammation, Wallerian degeneration^89–91^), inducing changes to morphology and functional capacity of regions distal to the injury^92–95^. For example, recent studies have shown that the accumulation of microvascular injuries, reflected in lower tissue volumes, is linked to both aphasia and response to treatment^96,97^. Further, recent work illustrates that acceleration of age-related patterns of atrophy independently contributes to aphasia severity^98,99^. The patterns capitalized on by the CNN in our study may have reflected some of these factors. Indeed, meta-analysis revealed many saliency patterns were associated with findings from past morphometry studies, including studies on aging. Future work that includes normative data could more clearly disentangle age-related patterns of atrophy predictive of aphasia severity.

### Heterogeneity of morphometry patterns predictive of aphasia severity

Clustering saliency maps revealed that the relatively localized group saliency observed in individuals with severe aphasia obscured a high degree of individual variability in integrity patterns exploited by the CNN (7 total). Clustering also revealed more precise morphometry patterns in nonsevere aphasia (6 total), which exhibited diffuse group saliency. Individuals were assigned to subgroups with high reliability, underscoring the robustness of the CNN. Lesion size and model error did not differ among subgroups, further stressing the degree of individualization based on global tissue integrity. That subgroups were more likely to show bilateral effects and sometimes exhibited lateralization patterns contradictory to their group-level trend indicated that any region could be important for predicting aphasia severity within individuals. The heterogeneity of integrity patterns associated with aphasia severity support a growing body of work characterizing this disorder as a complex of multidimensional deficits^100,101^ emerging from varied damage along large-scale networks^102^.

Meta-analytic decoding revealed that subgroups targeted different cognitive systems, although severe aphasia was more likely to be predicted when atrophy impacted regions supporting language. Only one nonsevere but four severe aphasia subgroups exhibited saliency associated with different language processes. Subgroups showed atrophy in different subsystems, including semantics when anterior temporal pole (TP) or posterior inferior temporal gyrus (pITG) were affected, reading when posterior temporoccipital cortex was affected, spontaneous overt speech when the middle frontal gyrus (MFG) was affected, and lexical-semantics when atrophy was widespread across the language network, including aforementioned temporal regions and additional regions in frontal cortex. Curiously, slightly different patterns of atrophy affecting largely the same swathe of frontal cortex in combination with the anterior TP were associated with higher-level cognition such as decision making, but also age, prior studies on morphology, and a range of neurodegenerative disorders. These patterns showed no association with language despite being predictive of severe and nonsevere aphasia, suggesting that chronic aphasia may interact with processes reflecting aging and neurodegeneration outside the language system in some patients but not others.

Implicated brain structures were consistent with prior work on the neurobiology of language. For example, subgroups involved frontal cortex, spanning the frontal pole (FP), frontal operculum (FO), inferior frontal gyrus (IFG) and MFG. Prior work in a similar capacity to ours has found that atrophy in IFG discriminates between healthy controls, stroke patients with aphasia and those without^17^. Impairments to different higher-level language functions are observed following damage to IFG^103,104^, damage to FO can result in aphasia^105^, and resection of MFG has been linked to apraxia of speech^106^. With respect to regions implicated in temporal cortex, atrophy in TP correlates with semantic dementia^107^, stimulation of pITG can disrupt naming^108^, and temporooccipital cortex supports reading^109^.

### Limitations

An important caveat to these results is that our data was downsampled to a lower resolution than typical to many neuroimaging analyses (though not voxel-based morphometry). Thus, it’s uncertain whether deep learning would be as effective for higher-dimensional neuroimaging data of the same kind. By the same token, our results do not suggest that deep learning would necessarily be as effective when applied to other neuroimaging modalities, patient populations, or behavioral measures. Future efforts investigating the impact of these factors on CNN performance could help inform researchers on the most appropriate tool for their task. Our results do not indicate that it would be impossible for classical machine learning to improve predictions of aphasia severity using features extracted by CNNs. Given the impossibility of testing every implementation of classical learning, we also cannot definitively say that classical machine learning cannot compete with CNNs for identification of severe aphasia. Our work simply demonstrates that CNNs can be highly effective for this task, more so than other commonly employed pipelines, by exploiting spatial dependencies. Finally, we have focused on segmented tissue maps to improve memory and computational efficiency for CNN training. Future work is likely to find better performance through utilization of more granular brain and behavioral data, as well as the integration of multiple imaging modalities^22^.

### Compliance with recommendations for machine-learning-related research

This study was conducted following transparent reporting of a multivariable prediction model for individual prognosis or diagnosis (TRIPOD) checklist included as supplementary Table S4.

## Supplementary Material

Supplement 1

## Figures and Tables

**Figure 3.**
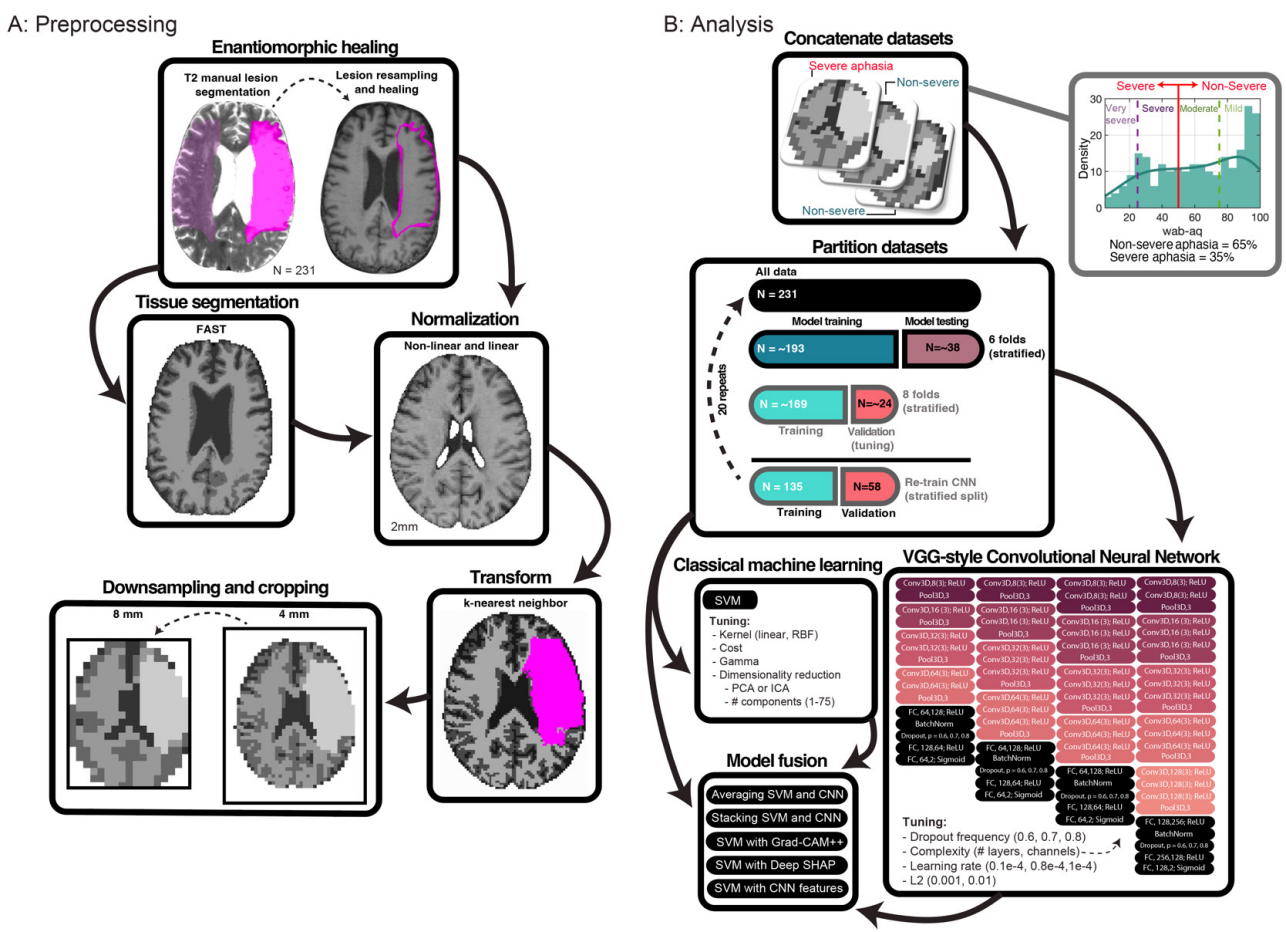
Overview of data preprocessing and analysis. **Panel A:** Lesion masks (opaque pink; left image) were manually drawn on 231 native T2 scans, resampled to native T1 scans (pink outline right image), refined, and healed by filling them with intact tissue around the homologues (c.f., opaque and transparent pink in image on left; result on right). Middle boxes: Cerebrospinal fluid, white, and grey matter tissues were segmented from healed T1s using FAST (left image). Healed T1s were registered to 2mm MNI template with FNIRT (right image). Bottom boxes: Tissue and lesion maps were normalized and combined, with lesions superseding other tissue (right image). Volumes were downsampled to 8mm and cropped (left image). **Panel B:** Volumes were concatenated across participants and linked to WAB-AQ (top black box), used to form “severe” (35%) and “nonsevere” (65%) aphasia categories by abridging very severe/severe and moderate/mild categories (denoted by vertical lines on histogram). Partitioning used a stratified, repeated, nested cross-validation scheme with 6 outer and 8 inner folds (middle box). For the CNN, the outer training dataset was repartitioned to permit training evaluation. Same partitions were used to train a CNN, SVM, and to implement model fusion strategies (bottom boxes). CNN tuning involved selecting network complexity, dropout frequency, learning rate and L2-norm (right bottom box). SVM tuning involved selection of kernel, cost, gamma, dimensionality reduction technique, and number of dimensions. Model fusion entailed prediction averaging, stacking, and chaining CNN-based feature extraction with SVM-based prediction, either using learned lower-dimensional features or higher-dimensional saliency (SHAP or Grad-CAM++).

**Figure 3.**
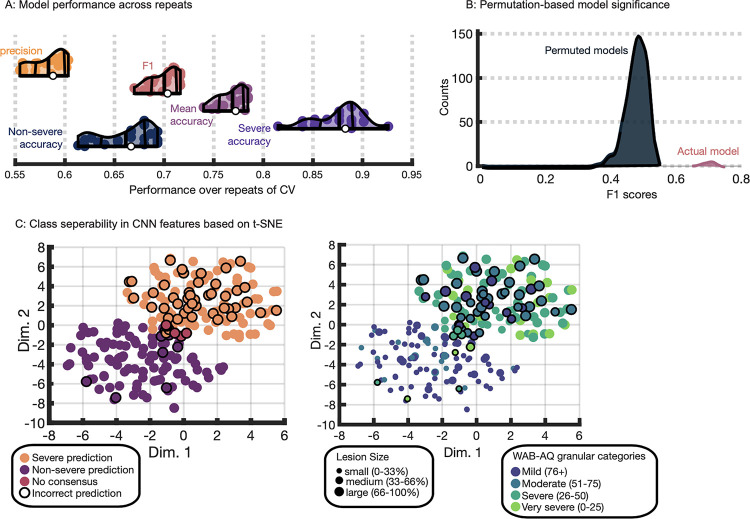
Convolutional neural network (CNN) performance. **Panel A:** Violin plots showing CNN performance over repeats of the nested cross-validation scheme. Colored dots represent the result of a single repeat. White dots represent median model performance according to a specific measure. Thick vertical lines inside each violin/density plot represent mean model performance. The flanking thinner, curved, vertical lines represent the interquartile range of performance. **Panel B:** The entire CNN model building procedure was repeated 500 times, each time permuting the class labels and recording the F1 score for the model during the testing phase. Permuted models’ F1 scores are described by the dark blue distribution and the pink distribution shows the unpermuted model F1 scores from panel A. **Panel C:** Both scatterplots show t-distributed stochastic neighborhood embeddings of the first fully connected layers of the CNNs (i.e., concatenating layers across all repeats and folds). Each dot represents a patient. In the left plot, the color of the dot represents the interpolated median prediction made by the CNNs across all repeats of the cross-validation scheme (in 4 patients interpolation did not identify consensus and these patients are designated by pink dots). In the right plot, dots are colored according to more granular WAB-AQ categories that our ‘severe’ and ‘nonsevere’ categories collapsed across. Brighter colors correspond to greater aphasia severity. Dot sizes represent relative lesion size using 3 quantile-based lesion size categories (smaller dots correspond to smaller lesions). In both scatterplots, incorrect predictions are distinguished by solid outlines.

## Data Availability

Data from the majority of participants analyzed here are publicly available as part of the Aphasia Recovery Cohort^110^: doi:10.18112/openneuro.ds004512.v2.0.0. A version of the dataset preprocessed in accordance with this manuscript have been made available here: 10.6084/m9.figshare.23579943. The full data used in the manuscript are available upon reasonable request to the corresponding author.
