# Supplementary material for: Distinct brain morphometry patterns revealed by deep learning improve prediction of aphasia severity
Source: Res Sq. 2023 Jul 3:rs.3.rs-3126126. Preprint. [Version 1] doi: 10.21203/rs.3.rs-3126126/v1 (PMC10350198; doi:10.21203/rs.3.rs-3126126/v1)
Supplement: Supplement 1 [file NIHPPRS3126126V1-supplement-1.pdf]

Supplemental material

Methods

Deep learning

Due to the computational expense associated with repeating the nested cross-validation procedure we used, the number of possible hyperparameter values was generally restricted to ~3, representing, for example, relatively high vs low regularization. We approximated the magnitude of the learning rates to use for tuning by experimenting with a random split of the concatenated training folds (70% training, 30% validation) and used the shallowest CNN architecture to find the best performing model from among a larger set of learning rates (0.1, 0.001, 0.0001, 0.00001, 0.000001, 0.0000001).

A series of metrics were tracked to initiate early stopping and avoid overfitting. The minimum loss on the validation dataset was used to select the trained model for testing. As long as the model had attained a minimum F1 score of 0.7 on the validation set, training was ceased if: i) training data was predicted with greater than 94% accuracy, ii) validation data was predicted with an F1 score above 0.9, iii) loss on the validation set had not improved at least once over the last 50 epochs, and iv) training and validation F1 scores were inversely correlated over the prior 30 epochs for 50 epochs in a row (i.e., had a Pearson correlation coefficient below -0.3; F1 scores were used instead of loss because they appeared to more clearly indicate when the model began overfitting).

Ensemble clustering

Internal validity of consensus matrices was estimated using the proportion of ambiguously clustered pairs (PAC) measure[73]. PAC is based on capturing the flatness of the mid-segment of a cumulative distribution function (CDF) curve generated for a consensus matrix by taking the fraction of sample pairs with consensus indices falling within some sub-interval (see steps 1 and 2 in panel E of Figure 3). The intuition behind this measure is that a clustering that is particularly reliable will be associated with a consensus matrix that is comprised largely of ones and zeros, reflecting the consistency with which sample pairs are either placed in the same cluster, or in different clusters, across subsamples. This kind of behavior within a consensus matrix is characterized by a CDF curve that has two large peaks around consensus values of one and zero, and a flat middle segment. Here, we use the common interval of [0.1,0.9] to compute PAC for each consensus matrix. We chose PAC because work has shown that this approach is more robust for determining the number of clusters in well-characterized datasets compared to other methods like qualitatively inspecting the "blockedness" of consensus heatmaps (i.e., heatmaps of consensus matrices), or looking for an elbow in the change of area under the CDF curve of consensus matrices when iteratively increasing the number of clusters[73]. Note that hereafter when we refer to the "reliability" of a parcellation, we refer to its PAC.

One issue with PAC that has only recently received attention is that it can show bias towards cluster models with a large number of clusters[111]. For example, in our experience it is common for PAC to show expected behavior as solution complexity increases until at some point, PAC begins to decrease, often monotonically. This behavior occurs because for very dense partitions with many clusters, consensus matrices are almost uniformly comprised of zeros, reflecting no consistent assignment of clusters across subsamples. The CDF curves for such matrices show a large peak at zero, sometimes a small peak around 1, and a relatively flat middle segment. The large amount of agreement about samples not belonging together artificially reduces PAC. We developed a method to identify consensus matrices that showed overall poor agreement despite increasing PAC by inspecting the extent to which consensus matrices were unimodal. The dip statistic was used to measure the unimodality of a consensus matrix by computing the difference between an empirical distribution function and the unimodal distribution function that minimizes this difference[112]. A p-value for the dip statistic, reflecting

whether a distribution is likely to be multimodal as opposed unimodal, was provided by comparing the obtained dip value with dip values calculated for 10,000 bootstrapped samples of the same size from a uniform distribution. Removing solutions with significantly unimodal consensus distributions allowed us to eliminate solutions with low PAC but poor consensus.

Affinity propagation

The procedure for affinity propagation involves passing two types of real-valued messages between data samples in order to group samples according to their most representative exemplar. Initially, all nodes are considered candidates for exemplars but over many iterations a number of core exemplars emerges. For each iteration of the message-passing process, responsibilities are sent from samples to potential exemplars to determine how well each exemplar is suited to represent the sender. Following this, availabilities are sent from the potential exemplars to samples in order to gather evidence for how appropriate it would be for each sample to choose this particular exemplar given the preferences of other samples. Availabilities and responsibilities between each pair of samples are initialized to zero and updated over iterations using a damping factor to control for the amount of influence exerted by update. The responsibility between sample i and potential exemplar k is defined as the similarity between these two samples minus the maximum of the sum of availability and similarity of all other potential exemplars to the same sample. Availability between potential exemplar k and sample i is defined as the minimum value between zero and the responsibility that exemplar k assigns to itself plus the sum of responsibilities that other samples assign to k (other than k or i). After availabilities and responsibilities are updated across some number of iterations, the final exemplars for each sample i are chosen based on which exemplar produces the highest sum of availability and responsibility for that sample. Typically, the number of clusters produced by affinity propagation is not explicitly chosen by the user. However, a preference value is assigned to each data sample by changing the diagonal on the similarity matrix, which allows for prior belief to influence which samples should serve as exemplars. Here, we used an implementation of affinity propagation that searches for an a priori defined number of clusters by iteratively changing the preference value using a bisection method[76].

ROI analysis of feature saliency

As with prior analyses, this one treated successful model predictions and thresholded negative SHAP values to zero prior to normalization. Perilesional ROIs were generated by applying mean dilation with a 3D box kernel of 10 mm to the lesion ROI, then masking the resulting map by the lesion ROI. Extralesional ROIs were created by including all voxels in the left hemisphere that were not contained in the lesion and perilesional ROIs. Homologue ROIs were generated by multiplying voxel coordinates in mm space by -1 (i.e., exploiting symmetry of the anatomical template).

Results

Tuning the SVM kernel function

Our comparisons between classical machine learning and deep learning used an optimistically biased estimate of SVM performance. This provided relatively more compelling evidence that SVMs underperformed compared to CNNs. An optimistic bound for SVM performance was estimated by training separate SVM models using linear and nonlinear kernel functions and retaining the best performing models for comparison to CNNs (see methods from main text). Here, we show that tuning the kernel function alongside other hyperparameters when building SVM models results in substantially worse performance than simply tuning the same parameters but holding the kernel function fixed to be a linear SVM (Figure S1). This was the case whether the SVM was trained on all features in the data, or if dimensionality reduction was

applied during training. When linear and nonlinear SVMs were trained independently, linear SVMs tended to outperform nonlinear SVMs. Note, performance for SVMs that were tuned for kernel function and did not involve dimensionality reduction as a preprocessing step was measured over 10 instead of 20 repeats of our cross-validation scheme (these models took the longest to train). However, the distribution of performance suggests a relatively consistent peak around 0.5 for F1 scores.

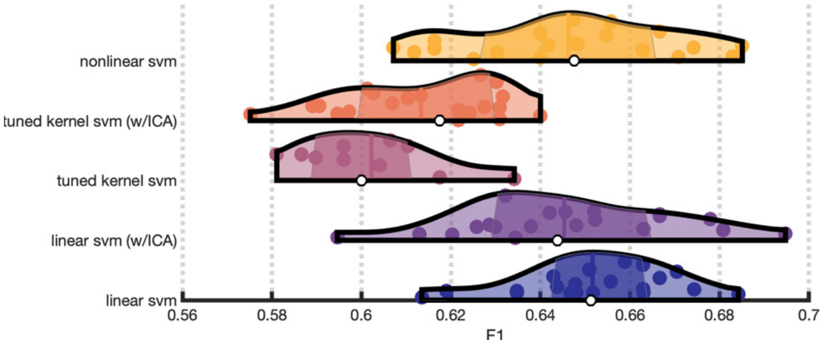

Figure S1. *Comparing SVM models with different kernel functions and tuning methods. The bottom two violin plots show data from the main text, reflecting SVM performance with a fixed linear kernel. In the bottom blue plot dimensionality reduction is not employed but in the purple plot above it, ICA is used. Including kernel function (i.e., linear and radial basis functions) as a hyperparameter lowers model performance. A SVM without dimensionality reduction performs worse when the kernel function is tuned (pink). A SVM with dimensionality reduction also performs relatively worse when the kernel function is tuned (orange). Finally, fixing the SVM to have a radial basis function kernel (nonlinear) also lowers SVM performance when dimensionality reduction is not used (yellow).*

t-SNE

We sought further evidence for CNN model robustness by verifying that it was able to learn consistent features across repeats of the cross-validation procedure. Feature consistency indicates the model is stable and can sufficiently capture underlying patterns without overfitting to the idiosyncrasies of specific training sets or fitting to noise or artifacts that may be present in the data. To this end, t-distributed stochastic neighbor embedding (t-SNE) was used to find a low-dimensional visualization of the features learned across all CNN models. Specifically, we combined models from the outer folds of all 20 cross-validation repeats and extracted their first fully connected layer activations, which act to map high-level features learned by the CNN on to a slightly lower dimensional representation for separating classes. If the CNNs had learned meaningful and consistent features, we expected to see class separation in the embedding. An optimal perplexity value for t-SNE was selected by evaluating solution divergence (Kullback-Leiber) across a large range of perplexity values. Generally, divergence decreases with perplexity so when this criterion is used for model selection, the highest tested perplexity value tends to be selected, favoring the most complex models. We weighted divergence by the magnitude of perplexity and used the pseudo BIC index to find the lowest perplexity value that minimized divergence, helping to balance t-SNE model complexity and accurate representation of the CNN features in an objective way (see [113] and https://github.com/alexteghipco/evalTSNE for implementation). Figure 3C (main text) shows clear and distinct clusters for different class predictions in the t-SNE embedding, confirming that the features learned by CNNs have a structure that exhibits consistency across repeats of cross validation. As t-SNE embedded samples based on an ensemble of CNN features, we determined the sample predictions of the CNN ensemble by taking the median prediction across all models (i.e., to verify that sample clusters in the embedding mapped onto class predictions). Class separation in the embedding

was weaker when considering true class labels instead of predicted labels, however, this fundamentally reflected the models' tendencies to trade-off more false positives for better severe aphasia prediction accuracy.

Evaluating the quality of CNN model predictions against classical machine learning Further, SVMs were substantially worse on severe class accuracy (M=0.7, SD=0.04) than CNNs (M=0.88, SD=0.03), $t(19) = -15.73$, $p < 0.00000001$, Cohen's $d = -3.52$. However, they had higher nonsevere accuracy (SVMs: M=0.76, SD=0.02; CNNs: M=0.66, SD=0.03), $t(19) = 12.34$, $p < 0.00000001$, Cohen's $d = 2.76$. Although SVMs did achieve significantly higher precision (M=0.61, SD=0.01) than CNNs (M=0.58, SD=0.01), $t(19) = -6$, $p < 0.00001$, Cohen's $d = 1.36$, CNN outperformance on F1 and mean accuracy measures was associated with larger effect sizes.

When dimensionality reduction was introduced, SVMs performed slightly worse on precision, $t(19) = -5$, $p < 0.0001$, Cohen's $d = -1.1$ (SVM: M=0.61, SD=0.01; SVM with dimensionality reduction: M=0.58, SD=0.03) and exhibited lower nonsevere class accuracy, $t(19) = -5.5$, $p < 0.0001$, Cohen's $d = -1.22$ (SVM: M=0.76, SD=0.02; SVM with dimensionality reduction: M=0.71, SD=0.02).

Fusing CNN and SVM predictions

Although stacked models attained higher nonsevere class accuracies (M=0.84, SD=0.02) than the CNNs (M=0.66, SD=0.03), $t(19) = 24.4$, $p < 0.00000001$, Cohen's $d = 5.45$, they also attained much lower severe class accuracies (M=0.57, SD=0.04) compared to the CNNs (M=0.88, SD=0.03), $t(19) = -32.3$, $p < 0.00000001$, Cohen's $d = 7.22$. For stacked models, a lower number of false positives for severity translated into higher precision (M=0.67, SD=0.03) compared to CNNs (M=0.58, SD=0.02), $t(19) = 10.8$, $p < 0.00000001$, Cohen's $d = 2.42$. Worse F1 scores and mean accuracies demonstrate stacking models does not improve predictions when balancing false positives and false negatives.

Training SVMs on CNN features

Although stacking did not produce better predictions (Figure 5, main text), it is possible that classical machine learning can improve overall model accuracy by more successfully exploiting features learned by the CNN. Further, it may be the case that PCA and ICA did not boost the performance of SVMs because the lower dimensional spaces produced by these methods did not capture the data as accurately (i.e., poorer SVM performance would still be attributable to the dimensionality of the data). If that were the case, we would expect SVMs to perform at least as well as CNNs when trained on CNN feature maps**.** Our results indicate this to be the case (Figure S2). Training SVMs on the CNN features embedded in the fully connected layers of the networks improves model performance relative to using PCA/ICA and results in SVMs that are roughly as good as CNNs at predicting aphasia severity. This in turn clarifies that SVMs were not better at exploiting the features learned by the networks. SVM with CNN features (M=0.69, SD=0.02) performed substantially better than SVM with ICA/PCA (M=0.65, SD=0.02) based on F1 scores, $t(19) = 8.8$, $p < 0.0000001$, Cohen's $d = 2.0204$. This was also the case for mean accuracy (SVM with CNN: M=0.77, SD=0.02; SVM with PCA/ICA: M=0.72, SD=0.02), $t(19) = 8.6$, $p < 0.0000001$, Cohen's $d = 2$. Moreover, there was no significant difference between F1 scores for CNN models and SVM models trained on CNN features ($p = 0.2$). There was also no significant difference between these models for mean accuracy ($p = 0.24$). However, SVMs trained with CNN features (M=0.7, SD=0.03) attained higher nonsevere class accuracy than CNNs (M=0.66, SD=0.03), $t(19) = 6.4$, $p < 0.00001$, Cohen's $d = 1.5$ but lower severe class accuracy than CNNs (SVMs with CNN features: M=0.83, SD=0.04; CNNs: M=0.88, SD=0.03), $t(19) = -5.72$, $p < 0.0001$, Cohen's $d = 1.3$. SVMs trained with CNN features (M=0.6, SD=0.02) also attained higher precision scores than CNNs (M=0.58, SD=0.02), $t(19) = 5.2$, $p < 0.00001$,

Cohen’s d = 1.2. As SVMs trained on CNN features did not improve the F1 score and decreased severe prediction accuracy, we interpret these results to suggest SVMs do not meaningfully improve on the CNN model.

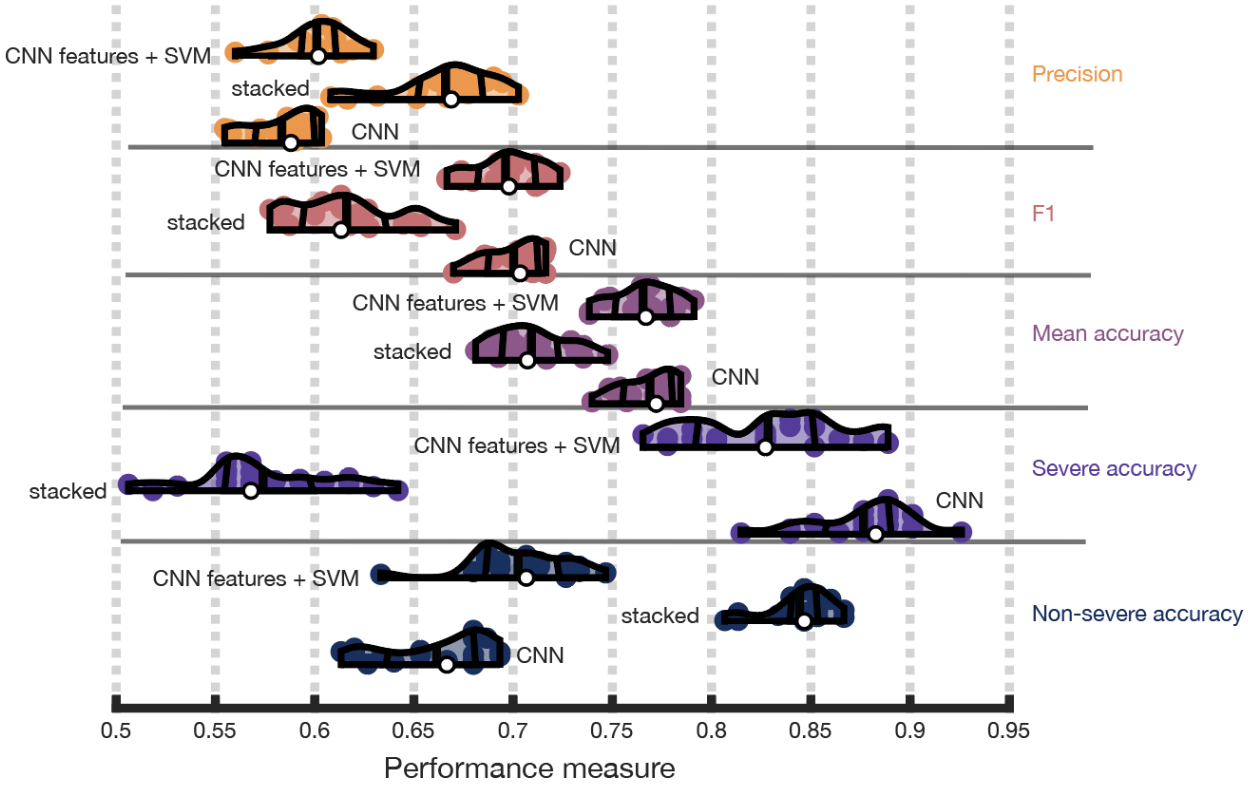

Figure S2. *SVM trained on CNN features. Violin plots showing CNN performance over 20 repeats of the cross-validation scheme relative to other strategies for fusing SVM and CNN predictions. Violin plot colors correspond to different performance measures which are additionally separated by horizontal lines. Within each performance measure the first or topmost violin plot shows the performance of a SVM trained on features extracted by a CNN (i.e., chaining CNN for feature extraction and SVM for prediction), the middle violin plot shows the performance of a tuned linear discriminant analysis model that stacks CNN and SVM prediction probabilities. The final violin plot shows CNN model performance as a baseline (i.e., from Figure 2).*

Example feature saliency maps for individuals

We expected deep SHAP maps generated for CNN and SHAP maps generated for SVM to share similarities, and to highlight very different trends compared to Grad-CAM++ maps generated for CNN. This finding would be consistent with the fact that Grad-CAM++ alone can recognize spatial dependencies exploited by the CNN. Figure S3 qualitatively illustrates this to be the case in a random sample of 6 participants that were correctly predicted by the CNN to have severe aphasia, and another 6 participants that were correctly predicted by the CNN to have nonsevere aphasia. Saliency maps were scaled for cleaner comparison of magnitude and relative importance. Note that Grad-CAM++ maps only represent feature importance for the predicted class while SHAP and deep SHAP maps represent feature importance that drives the model towards the predicted class (positive SHAP values) as well as the opposite class

(negative SHAP values). SHAP maps were scaled between -1 and 1 instead of 0 and 1 to preserve this signed information.

Across patients with different lesion sizes, the feature maps clearly demonstrated that Grad-CAM++ attributes successful prediction of severe aphasia by CNNs to features in the contralateral hemisphere, and attributes nonsevere prediction to ipsilateral features. Sometimes ipsilateral and contralateral features that were highlighted included the lesion, the perilesional area or their homologues. Although deep SHAP maps also explained CNN model predictions, they showed a vastly different spatial pattern, with both successful prediction of severe and nonsevere aphasia being attributed to features encompassing the lesion. Features that pulled predictions towards the opposite class appeared to highlight the intact portions of the perisylvian cortex, where lesion overlap across the cohort happened to be concentrated (see Figure 6 in main text). This suggested deep SHAP maps emphasized one of the multiple patterns exploited by the CNN: lesion size. SVM SHAP maps overall looked very similar to deep SHAP but severe aphasia predictions emphasized peripheral intralesional features whereas nonsevere aphasia predictions emphasized perilesional features. This more complex pattern implied that like the CNN, the SVM did not make predictions strictly based on lesion size. However, overall, it was much clearer from the feature maps that the CNN exploited information outside the lesion.

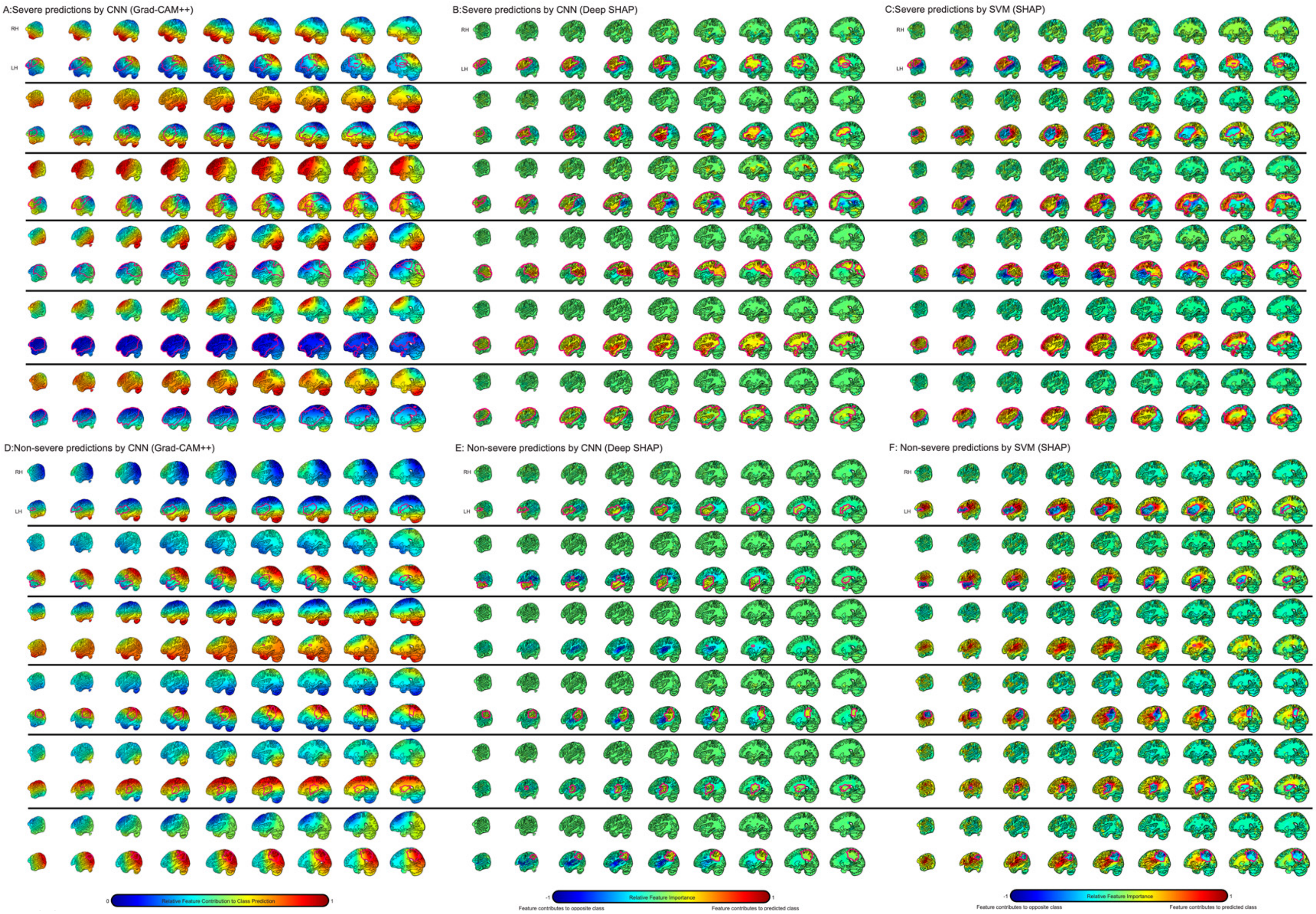

Figure S3. *Example feature saliency maps. Saliency maps that qualitatively highlight decision tendencies for the CNN and SVM models (i.e., across repeats). Each panel shows a montage of upsampled feature saliency maps (blue to red solid colors) and lesion masks (pink outline) overlayed on a normalized template (black outlines). Panels A, B and C show the same six example patients with severe aphasia and panels D, E and F show the same six example patients with nonsevere aphasia. Subjects with small, medium, and large lesions were randomly selected from a pool of samples correctly predicted by the CNN. Panels A, B, D, and E show saliency maps for the CNN. Panels C and F show saliency maps for SVM. Within each panel, rows as separated by a horizontal line represent unique example subjects.* ***Panel A:*** *Montage of upsampled Grad-CAM++ saliency maps for correct severe aphasia predictions by CNN. Hotter solid colors represent relatively higher feature importance and cooler solid colors represent relatively lower feature importance for the prediction (see colorbar for panel D).* ***Panel B:*** *Montage of upsampled Deep SHAP saliency maps for correct severe aphasia predictions by CNN. Hotter solid colors represent relatively higher feature importance for the (correct) class prediction and cooler solid colors represent relatively higher feature importance for the opposite (incorrect) class prediction (see colorbar for panel E).* ***Panel C:*** *Montage of upsampled SHAP saliency maps for correct severe aphasia predictions by SVM. Color coding of feature importance is consistent with panel B (also see colorbar for panel F). Note, the SVM made an incorrect prediction for the second participant, explaining the reverse spatial pattern.* ***Panel D:*** *Montage of upsampled Grad-CAM++ saliency maps for correct nonsevere aphasia predictions by CNN. Color coding of feature importance is consistent with the other panels.* ***Panel E:*** *Montage of upsampled Deep SHAP saliency maps for correct nonsevere aphasia predictions by CNN. Color coding of feature importance is consistent with the other panels.* ***Panel F:*** *Montage of upsampled SHAP saliency maps for correct nonsevere aphasia predictions by SVM. Color coding of feature importance is consistent with the other panels.*

Importance of bilateral features for CNN prediction

We verified that interhemispheric relationships in the Grad-CAM++ features were meaningful (e.g., homologues of the lesioned and perilesional area were often highlighted) by training SVMs independently on just the left and right hemisphere portions of the saliency maps (Figure S4). We found that using bilateral features resulted in higher F1 scores (M=0.69, SD=0.02) than restricting the analysis to right hemisphere features (M=0.66, SD=0.04), $t(19) = 5.15$, $p < 0.0001$, Cohen's $d = 1.2$. Bilateral features also resulted in higher F1 scores (M=0.69, SD=0.02) compared to left hemisphere features (M=0.67, SD=0.04), $t(19) = 3.1$, $p < 0.01$, Cohen's $d = 0.7$.

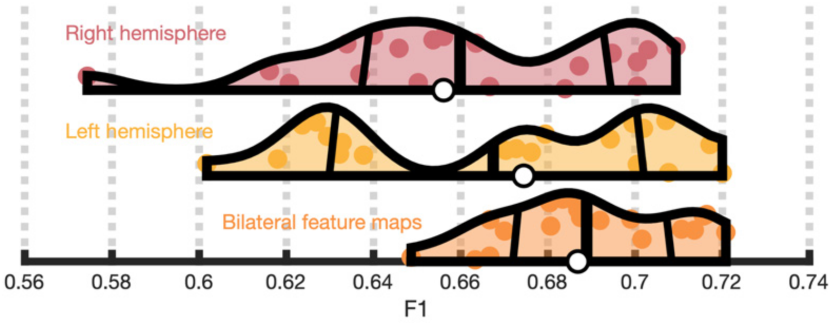

Figure S4. *SVM-based ablation experiments. Violin plots showing that a SVM trained on entire Grad-CAM++ saliency maps (orange) outperforms SVMs trained on just the left (yellow) or right hemisphere (pink) portions of the Grad-CAM++ maps according to the F1 score (x-axis).*

Model-order selection for unsupervised learning

Patients were clustered into subgroups with similar Grad-CAM++ maps using consensus clustering. A consensus matrix was generated for clustering solutions ranging from 3 to 30 clusters. The distribution of consensus values within each of these matrices are presented in Figure S5A and were used to guide model-order selection, the aim of which was to identify the most complex solution that remained reliable or stable, with high consensus across clustered subsamples of the dataset. Reliable solutions should be disproportionately made up of consensus values around 0 and 1, reflecting the fact that clustering always placed pairs of samples into the same or different clusters. The proportion of ambiguously clustered pairs (PAC) was used to assign each of these distributions a gross “reliability” value (see methods from supplemental material and main text) by capturing the flatness of a cumulative distribution function of consensus values within a consensus matrix. PAC across the range of investigated clustering solutions is presented in Figure S5B. PAC can be misleading when clustering mainly places pairs of samples into different clusters (i.e., shows mostly consensus values of 0 and not many consensus values of 1). Such solutions were eliminated from consideration by testing whether consensus distributions were significantly unimodal using Hartigan’s dip test (see opaque area in Figure S5). For severe patients, we selected the solution with the overall lowest PAC (i.e., 0.000015), which contained 7 clusters. For nonsevere patients, we selected the solution with the 3rd highest PAC, which was still remarkably low (0.005). Put another way, the selected solution showed ambiguity in cluster assignment for only 0.5% of samples in the data. Affinity propagation was used to extract the final clustering from the selected consensus matrices. We verified that affinity propagation could readily identify the structure of consensus matrices by repeating the clustering process 1000 times. We note that clusters were identical every single time affinity propagation was repeated. We also report that the exemplar samples around which affinity propagation organized clusters was highly consistent (Figure S5C). For severe patients, exemplars occasionally shifted between repeats of affinity propagation for some clusters. However, there was always a clear sample (i.e., patient) around which clusters formed across the majority of repeats.

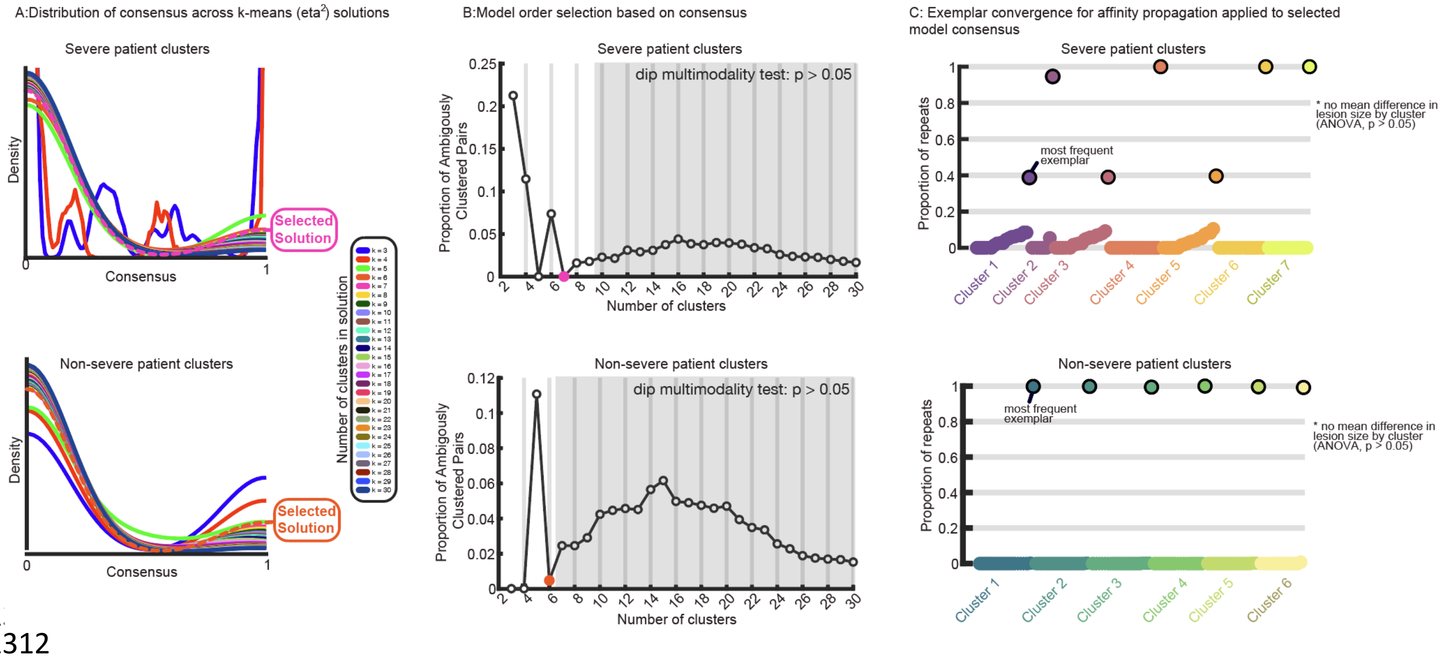

Figure S5. *Model-order selection for clustering Grad-CAM++ maps. Data for severe patients are shown in the top plot of each panel, and data for nonsevere patients are shown in the bottom plot.* ***Panel A:*** *Consensus matrices are plotted as distributions for solutions containing between 3 and 30 clusters. Each solution is assigned a unique color. The solution that was eventually selected is highlighted by a dotted line. Consensus values being plotted reflect the proportion of times a sample was assigned to the same cluster across subsamples of the data.* ***Panel B:*** *An empirical cumulative distribution function was generated for each consensus distribution from panel A. The difference in this function at consensus values of 0.9 and 0.1 were taken to generate the Proportion of Ambiguously Clustered Pairs (PAC). PAC values are plotted as a function of increasing solution complexity (i.e., higher number of clusters in the solution). An opaque area highlights distributions that were significantly unimodal according to Hartigan's dip test, and therefore were eliminated from consideration during model order selection. The selected solutions are highlighted by colored dots corresponding to the legend from panel A.* ***Panel C:*** *Exemplars for affinity propagation (AP), applied over the selected solutions' consensus matrix, were tracked over 1000 repeats of the AP clustering process. The proportion of times a certain sample was selected as the exemplar is shown on the y-axis and individual samples are shown on the x-axis, colored according to the cluster to which they were assigned by AP (cluster assignment was unanimous across repeats).*

Pearson correlation coefficients were estimated between Grad-CAM++ maps and sorted by patient subcategory to illustrate the extensive degree of within-subgroup similarity and between-subgroup dissimilarity. Further, correlation coefficients were divided into those measuring similarity between all patients within a subgroup, and those measuring similarity between patients within a subgroup and patients of other subgroups. The means of these correlation coefficients were used to probe within-subgroup and between-subgroup similarities (see Figure S6).

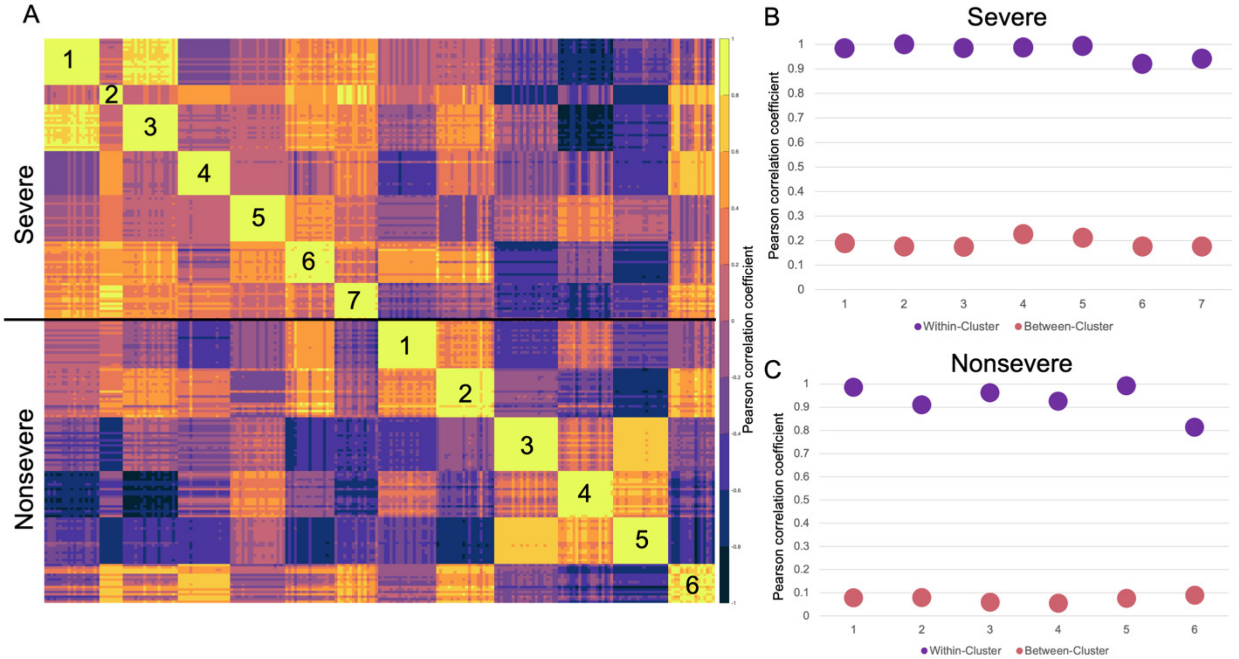

Figure S6. *Between and within-subgroup similarity.* ***Panel A:*** *Pearson correlation coefficients were computed between all patients' feature saliency maps, then patients were sorted by subgroup (i.e., clusters).* ***Panel B:*** *Mean correlation coefficients were computed within-*

subgroups and between-subgroups for severe patients. ***Panel C****: Identical to panel A but mean correlation coefficients were computed for nonsevere patients.*

Subtyping patterns learned by the CNN

Clustering revealed more diverse brain integrity patterns being exploited by the CNN than suggested by the relatively localized group-averaged saliency effects (Figure 9A; note all figure references in this section are for the main text). Consistent with the observation that left hemisphere regions still exhibited moderate feature importance (Figure 8), some severe aphasia patient subgroups showed saliency that peaked in the right hemisphere but was still relatively high in the left hemisphere (Figure 9B). As anticipated by the group map, most subgroups showed a strong peak in right anterior frontal cortex (subgroups 1,2,3,7). This peak was generally concentrated in a region spanning right middle frontal gyrus (MFG) and inferior frontal gyrus (IFG) (subgroups 1,2), however, some subgroups exhibited a peak in only one of these regions (MFG:7, IFG:3). One subgroup showed concomitant high saliency in the right temporal pole (TP) and slightly lower saliency in left MFG (subgroup 3). In contrast, other patients showed relatively high saliency in right IFG but had a stronger peak in right TP (subgroups 5 and 6). Patients showing this pattern could be subdivided further based on whether they additionally exhibited relatively higher feature importance in left parietal cortex (subgroup 5). The patient subgroups where feature saliency peaked in a region spanning right MFG and IFG could also be subdivided based on whether features in the left hemisphere were highlighted (subgroup 1), and whether the right posterior inferior temporal cortex (pITC) had moderately high importance (subgroup 2). Finally, one patient subgroup emphasized right occipital cortex (OC) but showed some feature importance in left anterior frontal and temporal cortex (subgroup 4). Notably, the patterns indicated by the exemplar participant around which each subgroup formed were remarkably similar to the other participants within that subgroup, corroborating the analysis of cluster consensus (Figure 9C).

To further understand the extent to which each subgroup reflected atrophy patterns affecting different brain systems, we decoded subgroup saliency maps as typified by the exemplar (see methods for more details). Decoding (Figure 9C) confirmed that most feature saliency patterns reflected language function (i.e., subgroups 2, 4, 5, and 7) but also underlined the extent to which they tapped into different language subsystems. For example, one subgroup showing more focal saliency around the right TP and left superior parietal cortex (SPC) was associated with semantics (i.e., top 3 terms mapping onto topic: semantic, word, knowledge; subgroup 5), whereas another subgroup (i.e., 2) showing saliency along large portions of frontal cortex and inferior temporal cortex (ITC) was associated with lexical-semantics, reading, comprehension, and pitch processing (i.e., topics: reading, phonological, readers; speech, auditory, temporal; words, word, lexical; verbs, verb, noun; language, sentences, comprehension; music, musical, pitch). Further, the subgroup with peak saliency in right OC (subgroup 4) displayed strongest associations to language, including reading and lexical processing (topics: hearing, deaf, sign; words, word, lexical; reading, phonological, readers; semantic, word, knowledge), but also tool knowledge (topic: tool, tools, knowledge) and sensory cortex (i.e., topic: visual, auditory, sensory). At the same, this subgroup displayed saliency associated with selective adaptation (topic: adaptation, selective, stimulus), as well as motion and object processing (topics: motion, mt, moving; object, objects, visual). A similar pattern of topics was associated with the subgroup exhibiting peak saliency around right MFG (i.e., subgroup 7), but also included associations to comprehension (i.e., topic: language, sentences, comprehension) and overt spontaneous speech (topic: verbal, fluency, overt). This subgroup was also associated with the dorsal and ventral streams despite being relatively focal (i.e., topic: dorsal, ventral, stream), as well as task switching, integration, item pairs, and action observation (topics: integration, process, task and task, switching, set; item, pairs, item; action, actions, observation). Curiously, several subgroups exhibited saliency related to other studies on

morphometry (topic: matter, gray, volume; subgroups 6) as well as age (i.e., topic: adolescents, adolescent, age; subgroups 1, 3, 6). These particular subgroups were also associated with a large number of topics representing demographics (e.g., sex), higher-level cognitive functions (e.g., decision making and social interactions), and a number of disorders (e.g., PTSD, AD, OCD, depression, epilepsy, ADHD, substance abuse, generic symptom severity, etc).

Despite diffuse feature importance observed at the group-level (Figure 10A), clustering of patients with nonsevere aphasia also highlighted diverse patterns of brain integrity learned by the CNN. Combined with the results of Figure 9, these patterns demonstrated that irrespective of aphasia severity, CNN predictions tended to revolve around the same anatomical regions, configured into slightly different networks, and with the hemisphere driving prediction generally varying as a function of severity. For example, a subgroup of patients with severe aphasia displayed highest feature saliency in right OC, while a subgroup of patients with nonsevere aphasia displayed peak saliency in left OC, but to a lesser extent also implicated portions of right OC (subgroup 4). A similar pattern, whereby the right hemisphere of a comparable network was emphasized for nonsevere patients, can be observed for a subgroup with feature saliency peaking in the TP and anterior frontal cortex (e.g., IFG; subgroup 2). Further, at least one saliency pattern indicated a shared network among aphasia subtypes, exhibiting peak saliency in right ITC but also high saliency in right MFG (subgroup 6). For patients with nonsevere aphasia, this network differed by having shifted distributions of peak saliency, showing relatively higher saliency in right ITC than MFG and additionally exhibited modest saliency in left MFG. Other subgroups had saliency maps that peaked around: i) just the left anterior TP, without additional peaks in anterior frontal cortex as observed for other subgroups (subgroup 1), ii) a region of the left SPC with slightly lower saliency extending anteriorly into motor cortex (subgroup 3), and iii) the same region of left SPC but with slightly lower saliency extending inferiorly into inferior parietal cortex (IPC) (subgroup 5). In multiple subgroups, right frontal (subgroup 1,6), right temporal (subgroup 2, 4,6) and right occipital cortex (subgroup 4) showed relatively moderate feature importance. Note, the subgroup that showed strongest right hemisphere lateralization of saliency exhibited high accuracy (Table S2).

Decoding of patient subtypes (Figure 10C) demonstrated that most patterns of morphometry associated with nonsevere aphasia were unrelated to language. Although many patients with nonsevere aphasia were grouped into subtypes that displayed peak saliency in regions commonly associated with language, only one subgroup (i.e., 6) showed a decoding pattern that mapped strongly onto language function (topics: semantic, word, knowledge; word, words, lexical). Coincidentally, this was the only subgroup exhibiting a right-lateralized saliency pattern. The saliency patterns of two subgroups (i.e., 1 and 2) were associated with morphometry studies (topics: matter, gray, volume) and one of these subgroups also displayed an association with age (topic: adolescents, adolescent, age; subgroups 2). Consistent to patients with severe aphasia that had similar topic associations, decoding also showed a strong relationship to demographics (e.g., sex), higher-level cognitive functions (e.g., emotional regulation, emotional processing, social interactions) and a number of disorders (e.g., PTSD, AD, OCD, depression, epilepsy, ADHD, substance abuse, schizophrenia, axnixety, BPD, generic symptom severity, etc.). This similarity between severe and nonsevere patient decodings is unsurprising, reflecting overlapping patterns of saliency despite different lateralizations The subgroup that demonstrated peak saliency in left OC (i.e., 4) decoded in a similar manner to the severe patient subgroup with peak saliency in right OC. Strong associations were observed with motion and object processing as well as visual processing more generally (topics: motion, mt, moving; object, objects, visual; blind, sighted, judgments; face, faces, fusiform; orientation, colour, separation; visual, auditory, sensory; spatial, location, space; perceptual, perception, visual; color, shape, shapes). The two subgroups with peak saliency favoring different aspects of SPC (i.e., subgroups 3 and 5) demonstrated somewhat different decoding patterns, although both were strongly associated with spatial attention,

working memory and reaction time (topics: spatial, location, space; attention, attention, visual; eye, gaze, saccade; wm, load, memory; response, time, reaction). The more anterior saliency pattern was uniquely associated with inhibition (topics: inhibition, response, inhibitory) while the posterior saliency pattern exhibited a broader decoding to unique topics. These included numerical processing, motion, imagery, hand movement, object recognition, and task training (topics: motion, mt, moving; imagery, mental, rotation; color, shapes, shape; ips, number, numerical; object, objects, visual; hand, movements, limb; bling, sighted, judgments; action, actions, observation, orientation, color, separation; movement, motor, movements; training, practice, trained, etc).

Studies using CNNs and neuroimaging in stroke patients

The main text cites and characterizes 19 PubMed studies that use CNNs and neuroimaging in stroke patients. A more detailed categorization of these studies is presented in a Sankey diagram (Figure S7) and the full list of studies can be found in Table S3. Note, of the 19 studies, one was a review of stroke segmentation studies, one included “stroke” in its abstract but treated Alzheimer’s patients, and one attempted identification of silent strokes from natural language processing of patient notes. In addition, 2 studies were focused on improving acquisition sequences with CNNs. Many of the studies for which the main goal was either identification or segmentation were not in stroke patients. These studies treated intracranial hemorrhages, arterial brain vessels, and microbleeds. A total of 13 studies treated stroke patients.

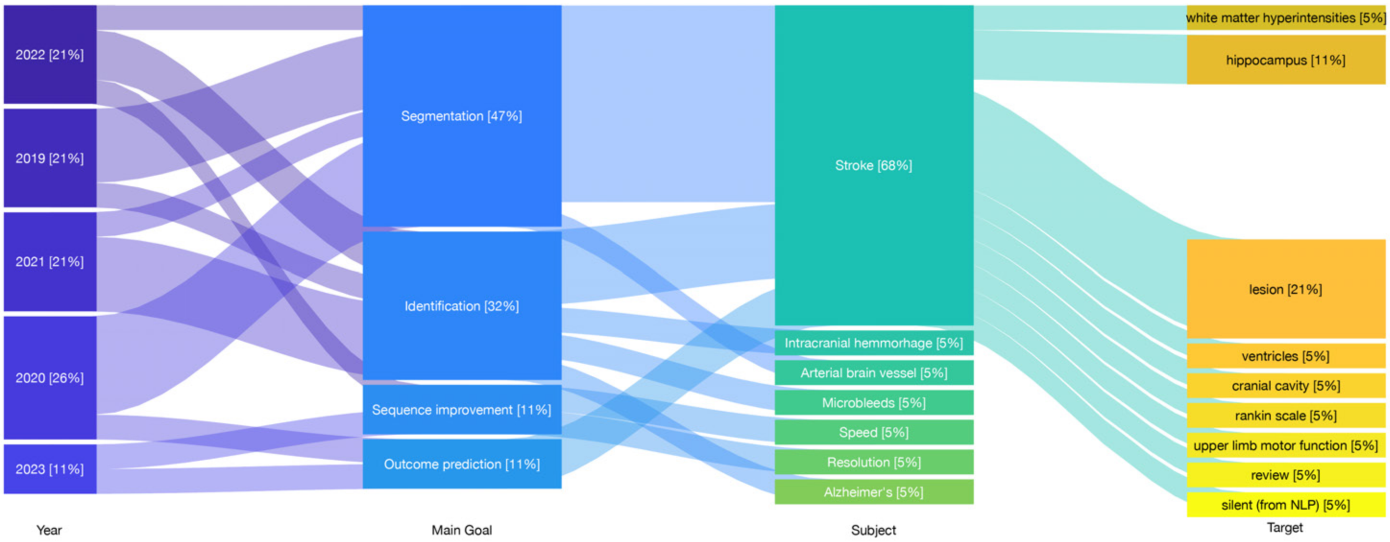

Figure S7. *Characterization of 19 studies retrieved from PubMed using CNNs and neuroimaging in stroke patients. From left to right, studies are categorized by year of publication (dark blue), their broad purpose for using a CNN (e.g., the CNN is used for segmentation; light blue), the specific subject to which they are applying the CNN (e.g., stroke patients; green), and the specific target the CNN is used to predict (e.g., lesions; yellow).*

Tables

Table S1: Lesion size and accuracy across severe patient subcategories

Subgroup	Lesion size	Accuracy	N
1	387.16	0.79	19
2	489	0.63	8
3	419.79	0.79	19
4	400.72	0.83	18
5	435.16	0.79	19
6	480.01	0.65	17
7	387.6	0.67	15

Table S2: Lesion size and accuracy across nonsevere patient subcategories

Subgroup	Lesion size	Accuracy	N
1	118.4	0.65	20
2	118.6	0.7	20
3	141.32	0.82	22
4	138.05	0.74	19
5	99.74	0.79	19
6	128	0.81	16

Table S3: Studies retrieved from PubMed using CNNs and neuroimaging in stroke patients

Study	Year	Main goal	Subject	Specific target
Forooshani et al.	2022	Segmentation	Stroke	white matter hyperintensities
Goubran et al.	2019	Segmentation	Stroke	hippocampus
Heit et al.	2021	Identification	Intracranial hemorrhage	Intracranial hemorrhage
Hilbert et al.	2020	Segmentation	Arterial brain vessel	Arterial brain vessel
Li et al.	2021	Identification	Microbleeds	Microbleeds
Meier et al.	2019	Segmentation	Stroke	lesion
Nitiri et al.	2021	Segmentation	Stroke	ventricles
Sheth et al.	2019	Segmentation	Stroke	lesion
Vass et al.	2022	Identification	Stroke	cranial cavity
Wu et al.	2020	Segmentation	Stroke	lesion
Xue et al.	2020	Segmentation	Stroke	lesion
Zavaliangos-Petropulu et al.	2022	Identification	Stroke	hippocampus
Duan et al.	2022	Sequence improvement	Speed	Speed
Iglesias et al.	2023	Sequence improvement	Resolution	Resolution

Nishi et al.	2020	Outcome prediction	Stroke	rankin scale
Karakis et al.	2023	Outcome prediction	Stroke	upper limb motor function
Karthik	2020	Segmentation	Stroke	review
Fu et al	2019	Identification	Stroke	silent (from NLP)
Dyrba et al	2021	Identification	Alzheimer's	Alzheimer's

Table S4: TRIPOD checklist for prediction model development (D) and validation (V).

Section/Topic	tem		Checklist Item	Page
**Title and abstract**				
Title	1	D;V	Identify the study as developing and/or validating a multivariable prediction model, the target population, and the outcome to be predicted.	1,4
Abstract	2	D;V	Provide a summary of objectives, study design, setting, participants, sample size, predictors, outcome, statistical analysis, results, and conclusions.	1
**Introduction**				
Background and objectives	3a	D;V	Explain the medical context (including whether diagnostic or prognostic) and rationale for developing or validating the multivariable prediction model, including references to existing models.	3-4
	3b	D;V	Specify the objectives, including whether the study describes the development or validation of the model or both.	4
**Methods**				
Source of data	4a	D;V	Describe the study design or source of data (e.g., randomized trial, cohort, or registry data), separately for the development and validation data sets, if applicable.	5-6
	4b	D;V	Specify the key study dates, including start of accrual; end of accrual; and, if applicable, end of follow-up.	5
Participants	5a	D;V	Specify key elements of the study setting (e.g., primary care, secondary care, general population) including number and location of centres.	5
	5b	D;V	Describe eligibility criteria for participants.	5
	5c	D;V	Give details of treatments received, if relevant.	-
Outcome	6a	D;V	Clearly define the outcome that is predicted by the prediction model, including how and when assessed.	5-8
	6b	D;V	Report any actions to blind assessment of the outcome to be predicted.	-
Predictors	7a	D;V	Clearly define all predictors used in developing or validating the multivariable prediction model, including how and when they were measured.	5-8
	7b	D;V	Report any actions to blind assessment of predictors for the outcome and other predictors.	-
Sample size	8	D;V	Explain how the study size was arrived at.	5
Missing data	9	D;V	Describe how missing data were handled (e.g., complete-case analysis, single imputation, multiple imputation) with details of any imputation method.	5
	0a	D	Describe how predictors were handled in the analyses.	5-8

Statistical analysis methods	0b	D	Specify type of model, all model-building procedures (including any predictor selection), and method for internal validation.	8-11
	0c	V	For validation, describe how the predictions were calculated.	7-11
	0d	D;V	Specify all measures used to assess model performance and, if relevant, to compare multiple models.	7-11
	0e	V	Describe any model updating (e.g., recalibration) arising from the validation, if done.	7-10
Risk groups	11	D;V	Provide details on how risk groups were created, if done.	-
Development vs. validation	12	V	For validation, identify any differences from the development data in setting, eligibility criteria, outcome, and predictors.	-
**Results**				
Participants	3a	D;V	Describe the flow of participants through the study, including the number of participants with and without the outcome and, if applicable, a summary of the follow-up time. A diagram may be helpful.	5
	3b	D;V	Describe the characteristics of the participants (basic demographics, clinical features, available predictors), including the number of participants with missing data for predictors and outcome.	5-7
	3c	V	For validation, show a comparison with the development data of the distribution of important variables (demographics, predictors and outcome).	7
Model development	4a	D	Specify the number of participants and outcome events in each analysis.	8-12
	4b	D	If done, report the unadjusted association between each candidate predictor and outcome.	-
Model specification	5a	D	Present the full prediction model to allow predictions for individuals (i.e., all regression coefficients, and model intercept or baseline survival at a given time point).	-
	5b	D	Explain how to the use the prediction model.	8-9
Model performance	16	D;V	Report performance measures (with CIs) for the prediction model.	Fig 3,4,5, 7
Model-updating	17	V	If done, report the results from any model updating (i.e., model specification, model performance).	-
**Discussion**				
Limitations	18	D;V	Discuss any limitations of the study (such as nonrepresentative sample, few events per predictor, missing data).	23
Interpretation	9a	V	For validation, discuss the results with reference to performance in the development data, and any other validation data.	-
	9b	D;V	Give an overall interpretation of the results, considering objectives, limitations, results from similar studies, and other relevant evidence.	20-23
Implications	20	D;V	Discuss the potential clinical use of the model and implications for future research.	8,21
**Other information**				
Supplementary information	21	D;V	Provide information about the availability of supplementary resources, such as study protocol, Web calculator, and data sets.	24

Funding	22	D;V	Give the source of funding and the role of the funders for the present study.	24
